# Hypermethylation of the non-imprinted maternal *MEG3* and paternal *MEST* alleles is highly variable among normal individuals

**DOI:** 10.1371/journal.pone.0184030

**Published:** 2017-08-30

**Authors:** Larissa Haertle, Anna Maierhofer, Julia Böck, Harald Lehnen, Yvonne Böttcher, Matthias Blüher, Martin Schorsch, Ramya Potabattula, Nady El Hajj, Silke Appenzeller, Thomas Haaf

**Affiliations:** 1 Institute of Human Genetics, Julius Maximilians University, Würzburg, Germany; 2 Department of Gynecology and Obstetrics, Municipal Clinics, Mönchengladbach, Germany; 3 Integrated Research and Treatment Center (IFB) Adiposity Diseases, Leipzig, Germany; 4 Institute of Clinical Medicine, University of Oslo, Lørenskog, Norway; 5 Fertility Center, Wiesbaden, Germany; 6 Core Unit Systems Medicine, Julius Maximilians University, Würzburg, Germany; 7 Comprehensive Cancer Center Mainfranken, Julius Maximilians University, Würzburg, Germany; University of Bristol, UNITED KINGDOM

## Abstract

Imprinted genes show parent-specific activity (functional haploidy), which makes them particularly vulnerable to epigenetic dysregulation. Here we studied the methylation profiles of oppositely imprinted genes at single DNA molecule resolution by two independent parental allele-specific deep bisulfite sequencing (DBS) techniques. Using Roche (GSJunior) next generation sequencing technology, we analyzed the maternally imprinted *MEST* promoter and the paternally imprinted *MEG3* intergenic (IG) differentially methylated region (DMR) in fetal cord blood, adult blood, and visceral adipose tissue. Epimutations were defined as paternal or maternal alleles with >50% aberrantly (de)methylated CpG sites, showing the wrong methylation imprint. The epimutation rates (range 2–66%) of the paternal *MEST* and the maternal *MEG3* IG DMR allele, which should be completely unmethylated, were significantly higher than those (0–15%) of the maternal *MEST* and paternal *MEG3* alleles, which are expected to be fully methylated. This hypermethylation of the non-imprinted allele (HNA) was independent of parental origin. Very low epimutation rates in sperm suggest that HNA occurred after fertilization. DBS with Illumina (MiSeq) technology confirmed HNA for the *MEST* promoter and the *MEG3* IG DMR, and to a lesser extent, for the paternally imprinted secondary *MEG3* promoter and the maternally imprinted *PEG3* promoter. HNA leads to biallelic methylation of imprinted genes in a considerable proportion of normal body cells (somatic mosaicism) and is highly variable between individuals. We propose that during development and differentiation maintenance of differential methylation at most imprinting control regions may become to some extent redundant. The accumulation of stochastic and environmentally-induced methylation errors on the non-imprinted allele may increase epigenetic diversity between cells and individuals.

## Introduction

Although it is generally assumed that the two alleles in a diploid genome are expressed to similar levels, 6–10% of all autosomal genes show monoallelic expression [1]. In most cases, silencing of one allele (allelic exclusion) occurs in a stochastic manner and different cells in a tissue randomly express either the maternal or the paternal copy. Only an estimated 100–200 imprinted genes (http://igc.otago.ac.nz/home.html) show parent-specific monoallelic expression, conferring an epigenetic asymmetry on the two parental genomes. In general, paternally expressed (maternally imprinted) genes such as the insulin-like growth factor 2 (*Igf2*) tend to enhance and maternally expressed (paternally imprinted) genes such as the Igf2 receptor (*Igf2r*) to restrict growth [2]. Apart from growth regulation, imprinted genes may be important for brain development [3] as well as behavioral and neurological functions after birth [4]. Most imprinted genes are located in clusters that contain differentially methylated regions (DMRs), which act as imprinting control regions (ICRs) [5]. Imprinted (i)DMRs are established in the male or female germline (primary DMRs), respectively, and/or during or shortly after fertilization (secondary DMRs). They escape genome-wide methylation reprogramming after fertilization and are stably inherited during somatic cell divisions [6,7]. In a narrow sense, imprinting disorders are a group of rare congenital syndromes which are caused by specific imprinting defects (failure of imprint establishment or maintenance), affecting growth, development, and metabolism [8]. Loss of imprinting (LOI) occurs in a large variety of human tumors and contributes to the multi-step process of tumorigenesis [9].

One important hallmark of DNA methylation patterns is their enormous plasticity. In addition to genome-wide epigenetic reprogramming in the germline and during preimplantation development [6,7], DNA methylation patterns are also influenced by environmental exposures [10,11]. According to the "developmental origins of health and disease (DOHAD)" or Barker hypothesis, adverse environmental exposures, in particular during periconceptional and/or intrauterine development confer life-long increased risks for metabolic and other complex diseases [12,13]. Persistent epigenetic changes are thought to be the mechanism by which environmental factors transmit risks to the exposed individual [14,15].

When studying the effects of environmental factors on the epigenome, usually the average methylation of a given locus is compared between exposed and non-exposed individuals. For example, previously we have shown that methylation of the mesoderm-specific transcript/paternally expressed gene 1 (*MEST*/*PEG1*) gene is slightly decreased in cord blood and placenta of children exposed to gestational diabetes mellitus [16]. However, since imprinted genes have one methylated and one unmethylated parental allele, the average methylation level (theoretically 50%) is a surrogate marker which is difficult to interpret. In addition, one cannot distinguish whether regional methylation changes are due to single CpG methylation errors in a large number of alleles (in a genomic DNA sample) or to a few allele methylation errors, where all or most CpGs in individual DNA molecules are aberrantly (de)methylated. Because it is usually the density of CpG methylation in a cis-regulatory region rather than individual CpGs that turns a gene "on" or "off” [17], single CpG methylation errors are most likely without functional consequences. In contrast, allele methylation errors must be considered as true epimutations, interfering with imprinted gene expression in the respective cell.

## Materials and methods

### Ethics statement

The Ethics Committee of the Medical Faculty at Würzburg University approved this study (votum no. 11/13 and 212/15). Written informed consent was obtained to use anonymized tissue samples for research purposes.

### Studied samples

Fetal cord blood (FCB) samples were collected at the Department of Gynecology and Obstetrics, Municipal Clinics, Mönchengladbach, adult blood (AB) at the Institute of Human Genetics, University of Würzburg, visceral adipose tissue (VAT) at the IFB Adiposity Diseases, University of Leipzig, and sperm at the Fertility Center, Wiesbaden, Germany. Genomic DNAs were isolated with the DNeasy Blood and Tissue Kit (Qiagen, Hilden, Germany) and bisulfite converted with the Epitect Bisulfite Kit (Qiagen). DNA quality and concentration were determined with a NanoDrop 2000c spectrometer (Thermo Fisher Scientific, Massachusetts, USA).

Informative samples for deep bisulfite sequencing (DBS) were selected by genotyping of single nucleotide polymorphisms (SNPs) rs7159412 (T/C, MAF 0.13) in the *MEG3* IG DMR, rs10134980 (A/C, MAF 0.17) in the *MEG3* promoter, rs2301335 (G/A, MAF 0.49) in *MEST*, and rs2302376 (C/T, MAF 0.20) in *PEG3*. Primers (S1 Table) were designed with Primer3 version 4.0.0 [18]. The PCR reaction mixtures consisted of 2.5 μl 10x PCR buffer with 20 mM MgCl_2_, 0.5 μl 10 mM PCR Grade Nucleotide Mix, 0.2 μl (5 U/μl) FastStart Taq DNA Polymerase (Roche Diagnostics, Mannheim, Germany), 1.0 μl (10 pmol/μl) of forward and reverse primers (Metabion, Martinsried, Germany), 18.8 μl PCR-grade water, and 1 μl (approximately 100 ng) of genomic DNA. Amplifications were performed with an initial denaturation step at 95°C for 5 min, 38 cycles of 95°C for 30 s, primer-specific annealing temperature (59°C for *MEG3* IG DMR, *MEG3* promoter, and *MEST*; 62°C for *PEG3*) for 30 s, and 72°C for 45 s, and a final extension step at 72°C for 10 min. Pyrosequencing was performed on a PyroMark Q96 MD pyrosequencing system with the PyroMark Gold Q96 CDT reagent kit and PyroMD software (Qiagen).

### DBS with Roche GSJunior

Primers (S2 Table) for sequencing bisulfite converted DNA were designed with the PyroMark Assay Design 2.0 software (Qiagen). Assays were established with the help of artificially methylated standard DNAs displaying 0%, 25%, 50%, 75%, and 100% methylation. For *MEST*, first-round PCR was performed in 25 μl reactions consisting of 12.5 μl HotStarTaq Master Mix (Qiagen), 0.5 μl (10 pmol/μl) of forward and reverse primers (Metabion), 10.5 μl PCR-grade water, and 1.0 μl (approximately 20 ng) bisulfite-converted template DNA. PCR was carried out with an initial denaturation step at 95°C for 15 min, 40 cycles of 95°C for 30 s, 54°C for 1 min, and 72°C for 1 min, and a final extension step at 72°C for 10 min. For the *MEG3* IG DMR, the 25 μl reaction mixture consisted of 2.5 μl 10 x PCR buffer, 4 μl of extra MgCl_2_ (25 mM), 0.5 μl (10 mM) PCR Grade Nucleotide Mix, 0.2 μl (5 U/μl) FastStart Taq DNA Polymerase (Roche Diagnostics), 0.5 μl (10 pmol/μl) of forward and reverse primers, 15.8 μl of PCR-grade water, and 1 μl template DNA. PCR was performed with denaturation at 95°C for 5 min, 50 cycles of 95°C for 30 s, 60°C for 30 s, and 72°C for 45 s, and final elongation at 72°C for 10 min. In the second-round PCR, sample-specific multiplex identifiers (MIDs), 454 Titanium A and B sequences, and key (TCAG) sequences were added. The 50 μl reaction mixture consisted of 25 μl HotStarTaq Master Mix, 1 μl (10 pmol/μl) of forward and reverse primers, 20 μl PCR-grate water, and 3 μl first-round PCR product. Each sample was amplified with a unique MID primer pair. After initial denaturation at 95°C for 10 min, 40 cycles of 95°C for 20 s and 72°C for 45 s (annealing and elongation), and a final elongation step at 72°C for 7 min were performed. The second-round amplification products were purified with Agencourt AMPure XP Beads (Beckmann Coulter, Krefeld, Germany). DNA quality was checked on the 2100 Bioanalyzer using DNA 7500 LabChips (Agilent Technologies, Waldbronn, Germany) and quantification performed with the NanoDrop 2000c spectrophotometer (Thermo Fisher Scientific). All samples were pooled in equimolar amounts and then diluted to a final concentration of 1x 10^6^ molecules/μl.

After emulsion PCR (emPCR), amplification products were sequenced on a Roche/454 GSJunior system, following the Roche emPCR Amplification Method and Sequencing Method Manual. Sequences of poor quality and mixed sequences, resulting from more than one bead in one well or several DNA fragments attached to one bead [19], were removed by the Roche Genome Sequencer software. Reads were aligned to genomic reference sequences and sorted by the sample-specific MIDs. Standard flowgram files (SFF) were analyzed with the Amplikyzer software [20], providing individual CpG site methylation over entire reads and samples.

### DBS with Illumina MiSeq

Bisulfite-converted DNA was amplified with amplicon-specific primers (S2 Table) in 50 μl PCR reactions, consisting of 5 μl 10 x PCR buffer with MgCl_2_ (20 mM), 1 μl PCR Grade Nucleotide Mix, 0.4 μl (5 U/μl) FastStart Taq DNA Polymerase (Roche Diagnostics), 2.0 μl (10 pmol/μl) of forward and reverse primers, 37.6 μl of PCR-grade water, and 2 μl template DNA. 1.6 μl of each primer and 38.4 μl PCR-grade water were used for the *MEG3* promoter. After an initial denaturation at 95°C for 5 min, 43 (for *MEG3* promoter and *PEG3*) and 45 cycles (*MEG3* IG DMR and *MEST*), respectively, were performed using 95°C for 30 s, primer-specific annealing temperature (53°C for *MEST*, *MEG3* IG DMR and promoter, and 57°C for *PEG3*) for 30 s, and 72°C for 60 s, and a final extension step at 72°C for 10 min. PCR products were purified with the QIAquick PCR Purification Kit (Qiagen) and quantified with the Qubit dsDNA BR Assay System (Life Technologies, Carlsbad, USA).

Samples were pooled in equimolar amounts with 24 index combinations and diluted to a final concentration of 0.2 ng/μl. Pools were purified with the Agencourt AMPure XP Beads (Beckmann Coulter). For adaptor ligation with NEBNext Multiplex Oligos for Illumina (Dual Index Primer Set 1; New England BioLabs, Frankfurt a. M., Germany), A-tailing was performed with Klenow Fragment (New England BioLabs) and ligation with T4 DNA Ligase (New England BioLabs), followed by another purification step. The optimal cycle number (ranging from 16 to 22) was determined with ligation efficiency PCR, using PfuTurbo Cx Hotstart DNA Polymerase (Agilent Technologies). During the final amplification step the pools were barcoded. After another purification, DNA concentration and fragment length were measured using the 2100 Bioanalyzer and the High Sensitivity DNA Kit (Agilent Technologies). For the final library, the 24 index pools were diluted to 4 nM and pooled. The resulting library was denatured, diluted to 12 pM, and mixed in a 5:1 ratio with 12 pM PhiX Control v3 (Illumina). Paired-end sequencing was performed on an Illumina MiSeq with a MiSeq Reagent Kit v3 (2 x 300 cycles) cartridge (Illumina, San Diego, USA).

After demultiplexing an initial quality assessment was performed with FastQC, v0.11.2 (http://www.bioinformatics.babraham.ac.uk/projects/fastqc/). Adapters and low quality reads were trimmed with TrimGalore, v0.4.0 (http://www.bioinformatics.babraham.ac.uk/projects/trim_galore/) powered by Cutadapt, v1.6 (https://cutadapt.readthedocs.io/en/stable/) [21]. TrimGalore parameters were set at:—paired,—trim1, -q 30,—length 50, -e 0. Trimmed paired reads were joined with the fastq-join option of ea-utils, v1.1.2–537 (https://expressionanalysis.github.io/ea-utils/) using the parameters–m 10 and–p 0. The reads were aligned to allele-specific human hg19 reference sequences with Bismark, v0.14.3 [22] and Bowtie2, v2.2.6 [23] with the parameters -n 1,—non_directional,—un—multicore 24,—score_min L,0,-0.1,—rdg 10,3,—rfg 10.3 in effect. Read alignments were processed with SAMtools v1.3 [24]. For methylation calling, the bismark_methylation_extractor was run with the parameters—comprehensive and—single-end. Alignments were visualized with the Integrative Genomics Viewer [25,26]. After sorting the samples by average allele-specific methylation, heatmaps were generated using the heatmap.2 function in the gplots R package with the cluster method “complete” and the distance method “euclidian”.

### Reverse transcription quantitative real-time PCR (RT-qPCR)

Primers for RT-qPCR (S3 Table) were designed with Primer3Plus [18]. RNA was isolated from VAT using the RNeasy Lipid Tissue Mini Kit (Qiagen); cDNA was synthesized from 2 μg RNA with the SuperScript II Reverse Transcriptase (Thermo Fisher Scientific). The RT-qPCR reaction volume (10 μl) consisted of 6.75 μl PCR-grade water, 2 μl 5x HOT FIREPol EvaGreen qPCR Mix Plus (Solis BioDyne, Tartu, Estonia), and 1 μl forward and reverse primer mix (3.3 pmol/μl) and 0.25 μl cDNA as template. An initial denaturing step of 5 min at 95°C was followed by 40 cycles of 15 s at 95°C, 20 s at 60°C, and 20 s at 72°C (melt curve: 60°C to 95°C). RT-qPCR was performed on ABI Viia 7 System (Applied Biosystems). Evaluation of melt curve and amplification plots was done with the QuantStudio Real-Time PCR Software v1.2.4 (Applied Biosystems) using the ΔΔCt method. Each sample was analyzed in technical triplets. Negative controls without cDNA template were used in each run for each primer pair. *GAPDH*, *HPRT1*, *IPO8*, and *RPLP0* [27] were used as reference genes for normalization.

### Statistical analyses

Both descriptive and inferential statistical analyses were performed with IBM SPSS version 23 (http://www.spss.com). For group comparisons, depending on the data distribution either nonparametric Mann-Whitney U or parametric T tests were performed. A p-value <0.05 was considered as significant. Numerical values of all measurements (of DBS and expression analyes) and other relevant information of each studied sample are found in S4 Table.

## Results

DBS was used to assess parental allele-specific methylation patterns of oppositely imprinted genes. Using Roche GSJunior technology, we studied the promoter of the maternally imprinted (maternally methylated) *MEST* promoter and the intergenic (IG) DMR of the paternally imprinted (paternally methylated) maternally expressed gene 3 (*MEG3*) in FCB, AB, and VAT. The more advanced MiSeq technology was then used to confirm the *MEST* promoter and *MEG3* IG DMR results and to study two additional loci, namely the paternally imprinted *MEG3* promoter and the maternally imprinted *PEG3* promoter in FCB. To distinguish parental alleles, heterozygous samples were selected by genotyping informative SNPs in the regions of interest. Ideally, the SNP ratio that is the number of reads for one allele divided by the number of reads for the second allele should be one. SNP ratios deviating from one indicate preferential amplification of one parental allele.

### DBS with the Roche GSJunior

**Fig 1:**
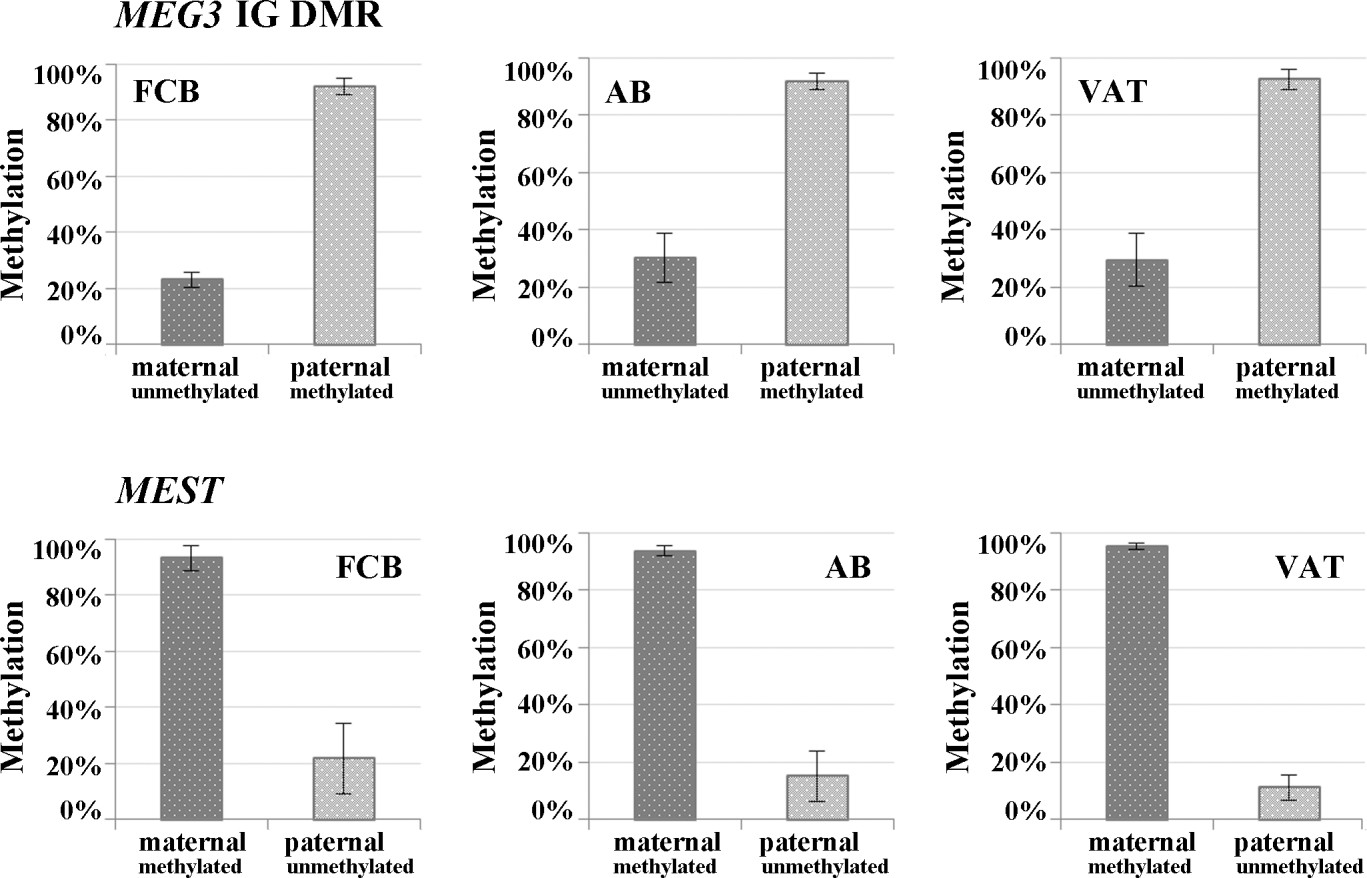
Parental allele-specific methylation of the *MEG3* IG DMR and the *MEST* promoter. Mean methylation levels and standard deviations of the paternal vs. maternal alleles were determined by DBS with the Roche GSJunior in FCB, AB, and VAT. For both genes, the non-imprinted allele, which is expected to be completely unmethylated, showed an aberrantly high methylation in all analyzed tissues, whereas the imprinted allele showed the expected (90–100%) methylation.

Altogether, 30 FCB, 23 AB, and 13 VAT samples were informative for the *MEG3* IG DMR and 50 FCB, 36 AB, and 24 VAT samples for *MEST*. An average of 796±261 reads and a SNP ratio of 0.78±0.14 per sample were obtained for the *MEG3* IG DMR and 1,036±355 reads and a SNP ratio of 0.72±0.2 for *MEST*, respectively. The methylated paternal allele of the *MEG3* IG DMR showed >90% methylation (Fig 1, upper panel), relatively close to the theoretical 100%. The maternal allele, which was expected to be completely unmethylated, showed considerable hypermethylation (23–30%) in all three analyzed tissues. Similar results were observed for the oppositely imprinted *MEST* promoter (Fig 1, bottom panel), where the unmethylated paternal allele displayed an excess methylation (11–22%).

**Fig 2:**
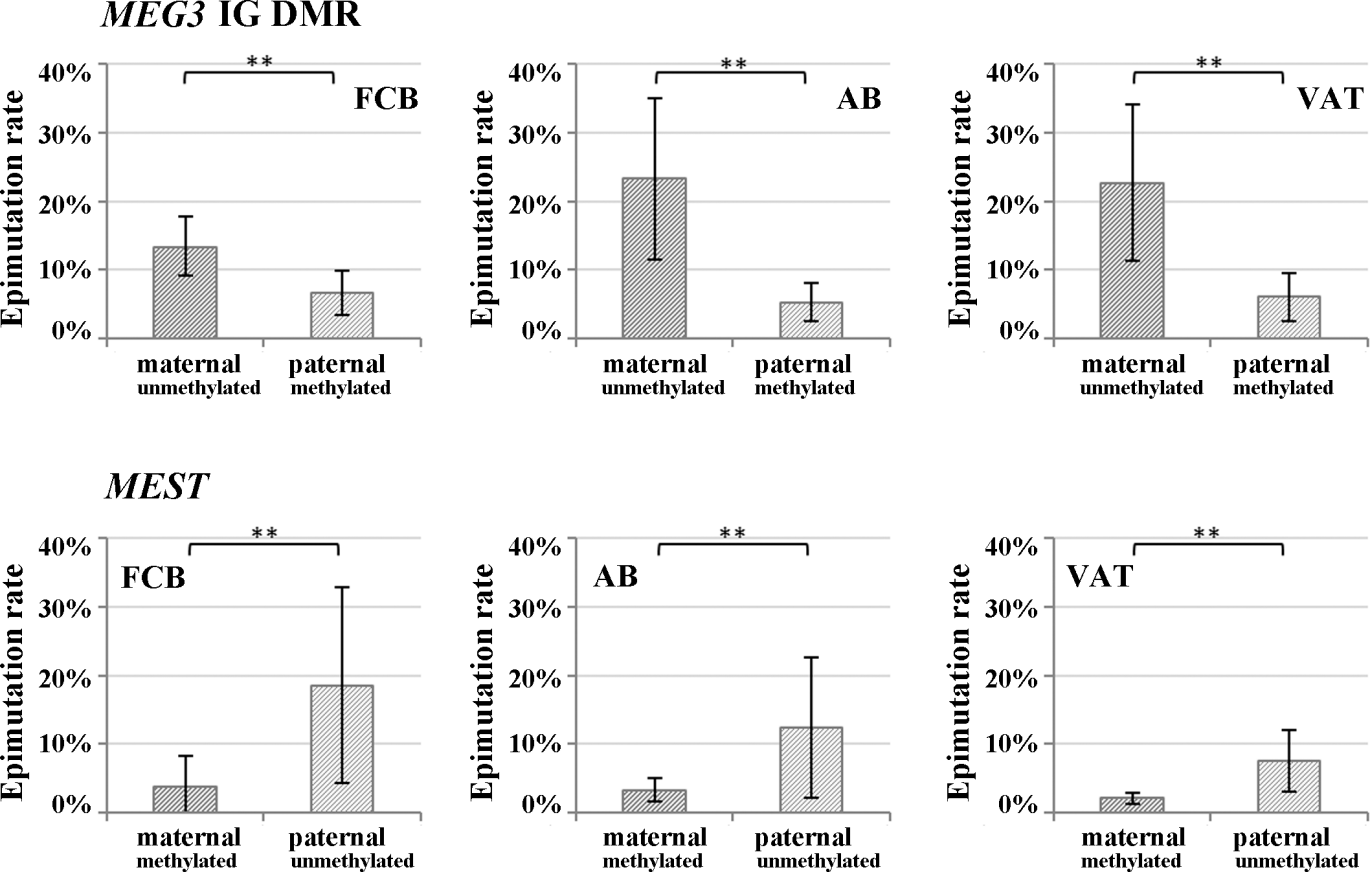
Parental allele-specific epimutation rates of the *MEG3* IG DMR and the *MEST* promoter. The percentage of alleles with >50% aberrantly (de)methylated CpGs was demined by DBS with the Roche GSJunior in FCB, AB, and VAT samples. The unmethylated alleles of the paternally imprinted *MEG3* and the maternally imprinted *MEST* genes displayed significantly higher epimutation rates than the methylated alleles.

**Fig 3:**
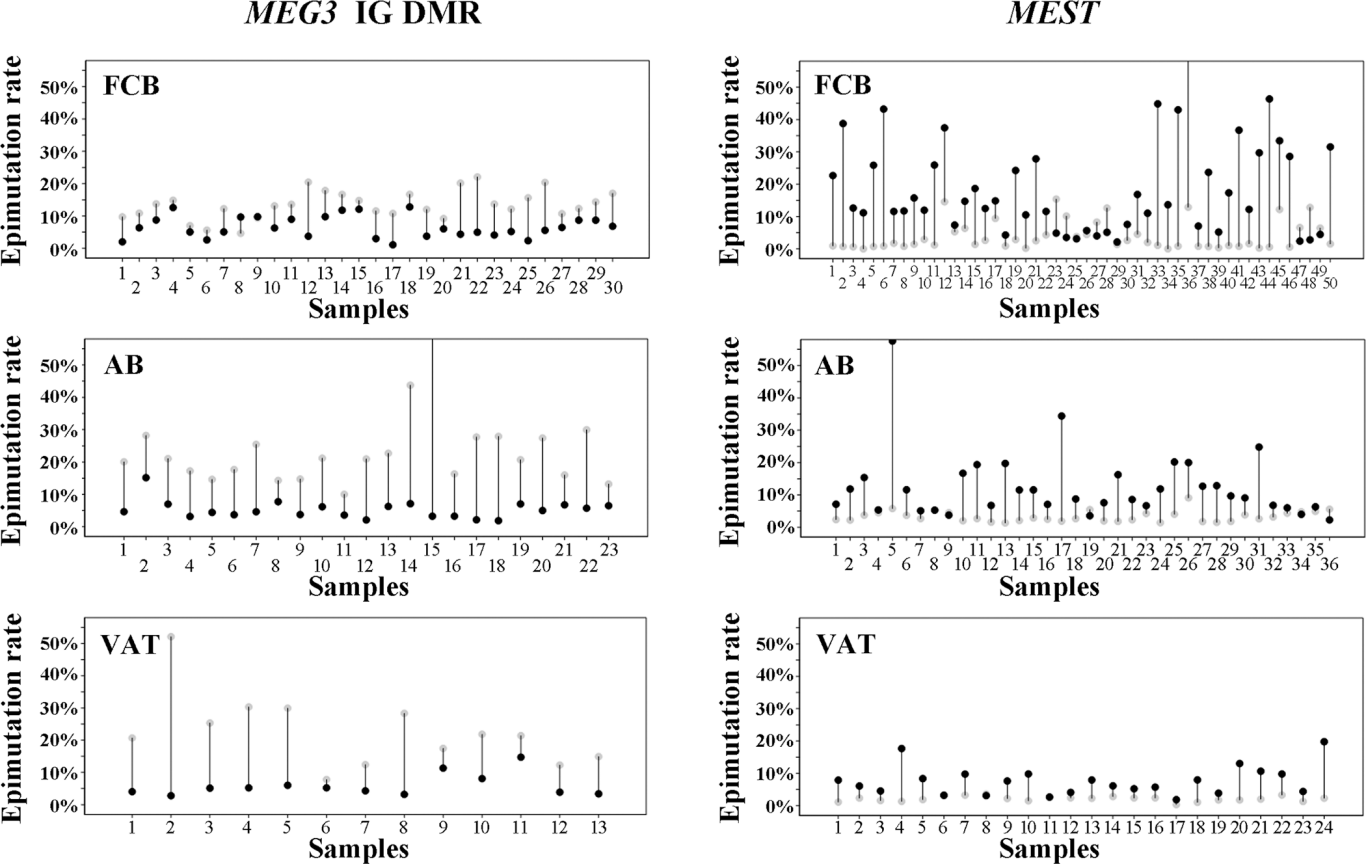
Epimutation rates of the *MEG3* IG DMR and the *MEST* promoter in individual samples. The percentage of paternal (black dots) versus maternal alleles (gray dots) with >50% aberrantly (de)methylated CpGs was determined by DBS with the Roche GSJunior in individual FCB, AB, and VAT samples. With very few exceptions, the unmethylated alleles of the paternally imprinted *MEG3* and the maternally imprinted *MEST* genes displayed much higher epimutation rates than the methylated alleles.

When looking at individual DNA molecules, the majority of the non-imprinted maternal *MEG3* IG DMR and paternal *MEST* alleles were unmethylated (0–20% methylation), however the observed allele methylation values covered the whole range from 0% to 100% with a high number of alleles displaying >50% methylation (S1A Fig). In contrast, the vast majority of the imprinted paternal *MEG3* and maternal *MEST* alleles were fully methylated (81–100% methylation) with a low number of alleles in the 0–80% methylation range. Alleles with >50% aberrantly (de)methylated CpGs were classified as epimutations. Epimutations were 3-4-fold more frequent on the normally unmethylated maternal *MEG3* (13–23%) and paternal *MEST* alleles (8–19%), compared to the normally methylated paternal *MEG3* (6–7%) and maternal *MEST* alleles (2–4%) (Fig 2; S5 Table). Irrespective of parental origin, the epimutation rate (ER) on the normally unmethylated non-imprinted allele was significantly (Mann-Whitney U test; p<0.001) higher than that on the normally methylated imprinted allele. Fig 3 displays the *MEG3* IG DMR and *MEST* ERs of the paternal versus the maternal allele for each individual FCB, AB, and VAT sample. Despite considerable variation in allele-specific methylation between samples, the ER on the non-imprinted (unmethylated) maternal *MEG3* IG DMR and paternal *MEST* allele was consistently and with very few execeptions considerable higher than that on the imprinted (methylated) allele. In individual samples, the ERs of the unmethylated allele ranged from 2% to 66% and of the methylated allele from 0% to 15% (S5 Table).

The paternal and maternal alleles of iDMRs are expected to maintain their germline methylation patterns in somatic cells. As a reference for the ERs on the paternal allele we analyzed sperm samples. Unfortunately, oocytes as reference for the maternal allele were not available. It is striking that mean methylation of the methylated paternal *MEG3* allele varied only 2–3% between sperm and different somatic tissues, whereas methylation variation of the unmethylated paternal *MEST* allele was between 10% and 20% (S2 Fig). The sperm ER was 0.9±1.6% for the *MEG3* IG DMR and 0.5±1.4% for *MEST*. The mean ERs of the paternal allele in FCB, AB, and VAT were significantly higher (5–7% for *MEG3* IG DMR and 8–19% for *MEST*). Thus, compared to somatic cells, the methylation imprints of paternally and maternally imprinted genes are strictly controlled in sperm and, to the extent of present knowledge, also in oocytes. Published single molecule analyses [28,29] suggest an ER of approximately 3% for the unmethylated *MEG3* IG DMR in mature human oocytes (from assisted reproduction), compared to a mean ER of 13–23% of the maternal allele in the somatic tissues analyzed here.

### DBS with the Illumina MiSeq

**Fig 4:**
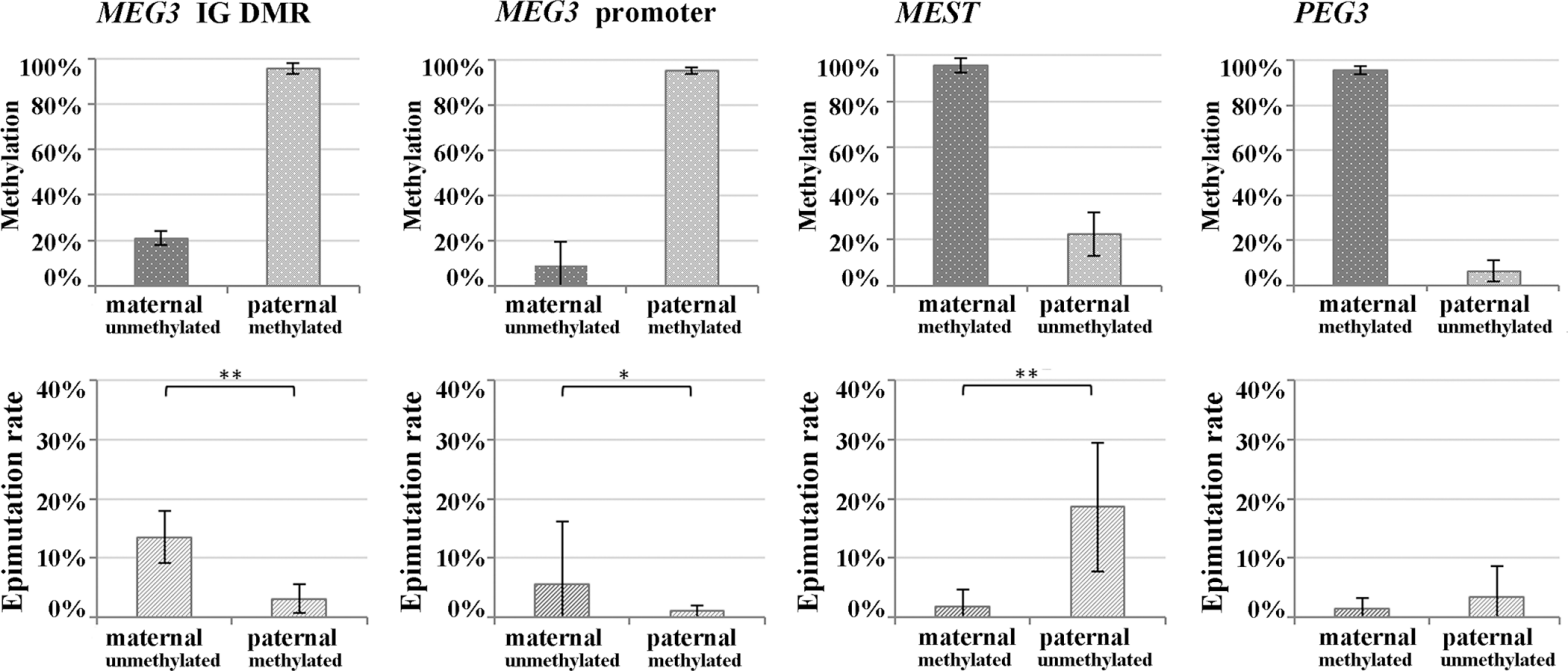
Mean FCB methylation and epimutation rates of maternal versus paternal alleles of the *MEG3* IG DMR, *MEG3* promoter, *MEST*, and *PEG3*. DBS was performed with the Illumina MiSeq. The non-imprinted allele of *MEG3* (both primary and secondary DMR) and *MEST* exhibited significantly higher epimutation rates than the imprinted allele.

To increase sequencing power, DBS protocols were adopted to the Illumina MiSeq. Forty-five informative FCB samples were analyzed for the *MEG3* IG DMR, 31 for the *MEG3* promoter DMR, 58 for *MEST*, and 21 for *PEG3*. The mean read number per sample was 46,836±28,743 for the *MEG3* IG DMR, 13,116±6,593 for the *MEG3* promoter, 19,928±19,771 for *MEST*, and 42,429±17,053 for *PEG3*. The corresponding SNP ratios were 0.71±0.12, 0.58±0.21, 0.71±0.15, and 0.60±0.13. Both the primary *MEG3* IG DMR and the secondary *MEG3* promoter DMR are paternally imprinted. Consistent with the Roche GSJunior data set (Fig 1), the paternal *MEG3* IG DMR showed 95.5±2.2% and the non-imprinted maternal allele 21.1±3.1% methylation (Fig 4, upper panel). For the secondary *MEG3* promoter DMR, paternal versus maternal allele methylation was 94.8±1.4% and 9.0± 10.6%, indicating that the demethylated state of the maternal allele is more strictly maintained than for the primary IG DMR. The maternally imprinted genes *MEST* and *PEG3* displayed 95.7±3.1% and 95.6±1.8% methylation on the maternal allele and 22.3±9.4% and 6.3±4.7% on the non-imprinted paternal allele.

For the *MEG3* IG DMR and *MEST* the distribution of single molecule methylation levels generated with Roche GSJunior and the MiSeq technology were virtually identical (S1 Fig), revealing a broad range (20–100%) of hypermethylated non-imprinted alleles, whereas the fully (81–100%) methylated state of the imprinted allele was more strictly maintained. For the secondary *MEG3* promoter and *PEG3* the number of hypermethylated non-imprinted alleles was much lower than for the *MEG3* IG DMR and the *MEST* promoter. For the *MEG3* IG DMR, the *MEG3* promoter, and *MEST*, the ER on the non-imprinted (unmethylated) allele was significantly (p<0.001) higher than on the imprinted (methylated) allele (Fig 4, lower panel). The secondary *MEG3* promoter DMR exhibited significantly (p<0.001) lower ERs on both the non-imprinted and imprinted allele than the primary *MEG3* IG DMR.

### Expression analysis

To test possible regulatory effects of hypermethylation of the non-imprinted allele (HNA), *MEST* and *MEG3* expression were quantified by RT-qPCR in 20 and 7 VAT samples, respectively. Unfortunately, these were the only samples available for RNA analyses. The difference between the ERs on the non-imprinted and the imprinted parental allele (ER_NI_-ER_I_) corresponds to the percentage of cells with biallelic methylation. The expression of *MEST* assays 1 and 2 (both targeting isoform 2) correlated with each other and were highly variable among the studied samples (S3 Fig). Unexpectedly, there was a positive correlation (Spearman rho = 0.5; p = 0.03 and 0.02 for assay 1 and 2, respectively) between methylation and expression. In addition, expression was positively correlated with BMI (rho = 0.4; p = 0.11 and 0.05, respectively), ranging from 21 to 54 kg/m^2^ in the analyzed individuals. For the *MEG3* IG DMR, expression and methylation appeared to be inversely correlated, but due to the low sample size the results were not significant.

## Discussion

### DBS for the study of imprinted genes

DBS including a user-friendly analysis software was first established on the Roche GSJunior [20,30], yielding approximately 1000 reads per gene and sample. DBS with the Illumina MiSeq increased the average read number to 20,000–40,000. The developed protocols for library preparation and data analysis can be relatively easily adopted to high-throughput Illumina sequencers, which will further increase power (number of genes analyzed simultaneously, number of reads). Single molecule methylation analysis by DBS provides novel insights into imprinting mechanisms. Combined with SNP typing, it allows one to determine the methylation profiles of several thousand individual paternal versus maternal alleles in larger numbers of samples.

For most (but not all) iDMRs, the allele-specific methylation patterns were not strictly maintained in somatic tissues and the non-imprinted alleles were more susceptible to epimutations. A considerable number of blood and fat samples from normal individuals displayed ERs of the non-imprinted *MEG3* IG DMR and *MEST* alleles between 30% and 60%. Although minor differences in cell composition may contribute to methylation variation between tissue samples (blood and VAT, respectively), they cannot account for the observed huge range (from 2% to 66%) in HNA between individuals. The results were highly consistent, using different library preparation and NGS techniques (Roche and Illumina). This largely excludes experimental or bioinformatical artefacts. Direct measurement of ERs of the non-imprinted versus the imprinted allele is technically challenging. The sequence divergence between methylated and unmethylated molecules (of the same allele) after bisulfite conversion may lead to an amplification bias towards one, i.e. the unmethylated T-rich product [31]. In addition, DNA molecules with incomplete bisulfite conversion may mimick hypermethylated alleles. Reduced sequence complexity, asymmetric C to T alignments, and increased searching space, compared to the original reference sequence, can result in false-positive matches [32]. To overcome these problems, we excluded all reads with low sequencing quality or sequencing errors. However, improved protocols with single molecule tagging before PCR amplification may be necessary to exclude any amplification bias with 100% confidence.

### Hypermethylation of the non-imprinted allele (HNA)

In this study we assessed the allele-specific methylation profile of four iDMRs at single molecule resolution. Only the *PEG3* promoter displayed the expected black and white pattern of allele-specific methylation, with comparably low (1.5% and 3.4%) ERs on both parental alleles. The maternally imprinted *MEST* promoter and the paternally imprinted *MEG3* IG DMR showed significantly higher ERs on the unmethylated allele than on the methylated allele. The secondary *MEG3* promoter DMR showed an intermediate pattern. Although the ER was significantly higher on the non-imprinted maternal allele, its unmethylated state was much better maintained than for the *MEG3* IG DMR.

Classical plasmid cloning studies [28] revealed that the methylation imprint of the *MEG3* IG DMR is strictly maintained in oocytes (fully demethylated) and sperm (fully methylated), whereas a considerable proportion of alleles in preimplantation embryos and somatic tissues (amniocytes, fetal and adult blood) showed intermediate methylation patterns. Although parental alleles were not distinguished, it is plausible to assume that most intermediate patterns represented hypermethylated maternal alleles. Collectively, these results suggest that relaxation of the primary *MEG3* IG DMR occurs after imprinting of the secondary *MEG3* promoter DMR has been established in the early embryo. Then, the IG DMR, which functions as an upstream regulator, may at least partially become redundant.

Previous studies have shown loss/relaxation of imprinting (LOI/ROI) at the transcriptional level. Biallelic expression of *IGF2* was observed in 10–20% of blood samples from normal healthy individuals [33,34]. Reports that biallelic *IGF2* expression in normal colonic mucosa and blood was severalfold more frequent in colorectal cancer patients than in healthy individuals have promoted the idea that LOI predisposes to cancer [35,36]. Interestingly, cord bloods from healthy newborns did not show an association of LOI/ROI with increased *IGF2* mRNA levels or abnormal methylation levels [34]. Our results revealed an enormous variation of allele-specific methylation of at least some imprinted genes in normal tissues/individuals. In essentially all studied samples, the *MEG3* IG DMR and *MEST* ERs were severalfold higher on the unmethylated than on the methylated allele, implying biallelic methylation in a considerable number (up to 50%) of cells. Comparable HNA was observed in different somatic tissues (blood and fat) but not in germ cells and there was no obvious increase in the percentage of abnormally methylated alleles with age (between fetal cord blood and adult blood). Thus, HNA most likely originated during early somatic development (before separation of the different embryonal layers). Some iDMRs were more susceptible to HNA than others, irrespective of parental origin.

Intuitively, HNA of the studied iDMRs should be associated with transcriptional gene silencing. However, *MEST* showed a positive correlation between methylation and expression, indicating that its regulation in VAT is more complex. The human *MEST* gene is endowed with two promoters generating transcripts with alternative first exons. The promoter studied here is thought to control monoallelic expression of isoform 1, whereas the isoform 2 under control of the second promoter is biallelically expressed [37]. In addition to the imprinted isoform 1, the *MEST* iDMR controls the promoter of an antisense transcript. In a variety of tumors hypermethylation of this DMR was associated with upregulation of the sense gene, most likely by downregulation of the antisense transcript [38]. A similar mechanism may be responsible for *MEST* upregulation in fat tissue. It has been reported that the imprinted isoform 1 is upregulated in fat tissue of obese individuals in both humans and mice [39,40]. In the VAT samples studied here expression of isoform 2 also increased with BMI. In rodent models, *Mest* expression levels correlated with the development of diet-induced adiposity and fat mass expansion [41,42]. *Mest* upregulation and *MEST* hypomethylation have been linked to an early overnutritional environment in mice [43] and humans [16], respectively. Thus, *MEST* is a primary candidate for developmental programming of a metabolic phenotype(s).

The maternally expressed gene 3 (*MEG3*) is controlled by a primary germline DMR and a secondary DMR, which is established after fertilization. The primary *MEG3* IG DMR lies in the intergenic region 13 kb upstream and the secondary DMR in the *MEG3* promoter 1.5 kb upstream of the transcription start [44]. The maternal *MEG3* locus expresses a dozen or more alternatively spliced long non-coding RNAs as well as microRNAs and small nucleolar RNAs [45,46]. In VAT methylation of the *MEG3* IG DMR appeared to be associated with decreased expression, although the results were not significant.

### Imprinted genes, metastable epialleles, and developmental programming

During early development the epigenome is highly susceptible to environmental factors, i.e. assisted reproductive technologies [47,48] or maternal nutrition [14,15]. Imprinted genes may be particularly susceptible to adverse environmental exposures because they escape genome-wide epigenetic reprogramming after fertilization and are functionally haploid. To maintain functionally important parent-specific methylation patterns during many cell divisions in the developing embryo, the unmethylated parental allele must be protected very efficiently against de novo methylation. At the *H19*-*IGF2* locus, differential methylation is maintained by binding of CTCF zinc-finger proteins to the unmethylated allele [49,50]. In addition, histone proteins may be involved. Enrichment of H3K4me2 and H3K4me3 on the unmethylated parental chromosome may prevent binding of DNA methyltransferases and de novo methylation. Aberrantly acquired methylation marks may be enzymatically removed by ten-eleven translocation proteins [51]. Thus, maintenance of an unmethylated state at one parental allele requires an interplay of different mechanisms. Stochastic and/or environmentally induced errors in this complex process may lead to HNA during somatic development.

At first glance, it may be surprising that the iDMRs of *MEST* and *MEG3*, genes controlling somatic growth, display such enormous epigenetic variation. Previously, it has been shown that *IGF2/Igf2*, another imprinted region involved in growth regulation, shows considerable variation of allelic expression between normal individuals [33,34] and tissue-specific variation in allelic methylation during development [52]. Interestingly, growth-regulating genes, including *MEST* and *MEG3*, in an imprinted gene network become transcriptionally downregulated in multiple organs during postnatal growth deceleration/cessation [53]. Thus, HNA may be part of a physiological process for regulation of an imprinted gene network to limit body size. This process may be susceptible to developmental programming through environmental factors to enhance variation at the population level.

In many aspects HNA resembles metastable epialleles (ME), which can display variable methylation and expression in the absence of genetic variation and thus lead to phenotypic variation in genetically identical individuals [54]. ME candidates have also been identified in humans [55]. Epigenetic programming of MEs by environmental exposures such as maternal nutrition [56] and assisted reproduction [57] provides a link to common adult disease (DOHAD).

## Conclusions

Some growth-regulating imprinted genes such as *MEST* and *MEG3*, are susceptible to HNA during development and differentiation, whereas the iDMRs of others (i.e. *PEG3*) are strictly maintained. HNA leads to a variable number of cells with biallelic ICR methylation (somatic mosaicism). Similar to MEs, the extent of HNA is highly variable between individuals, most likely due to stochastic and environmental programming during early development, and may contribute an additional layer of complexity to phenotypic diversity in mammals.

## Supporting information

S1 Fig Methylation percentage distribution of non-imprinted (unmethylated) and imprinted (methylated) alleles. (PDF)

S2 Fig Mean methylation and epimutation rates of the paternal *MEG3* IG DMR and the *MEST* allele in sperm, compared to FCB, AB, and VAT. (PDF)

S3 Fig Relationship between methylation of the *MEST* promoter and the *MEG3* IG DMR and gene expression in VAT samples. (PDF)

S1 Table Pyrosequencing primers for genotyping. (PDF)

S2 Table Primers for deep bisulfite sequencing. (PDF)

S3 Table Primers for reverse transcription quantitative real-time PCR. (PDF)

S4 Table DBS and expression measurements of all analyzed samples. (PDF)

S5 Table Epimutation rates measured by DBS. (PDF)
