# Supplementary material for: Hypermethylation of the non-imprinted maternal MEG3 and paternal MEST alleles is highly variable among normal individuals
Source: PLoS One. 2017 Aug 30;12(8):e0184030. doi: 10.1371/journal.pone.0184030 (PMC5576652; doi:10.1371/journal.pone.0184030)
Supplement: S1 Fig — (PDF) [file pone.0184030.s001.pdf]

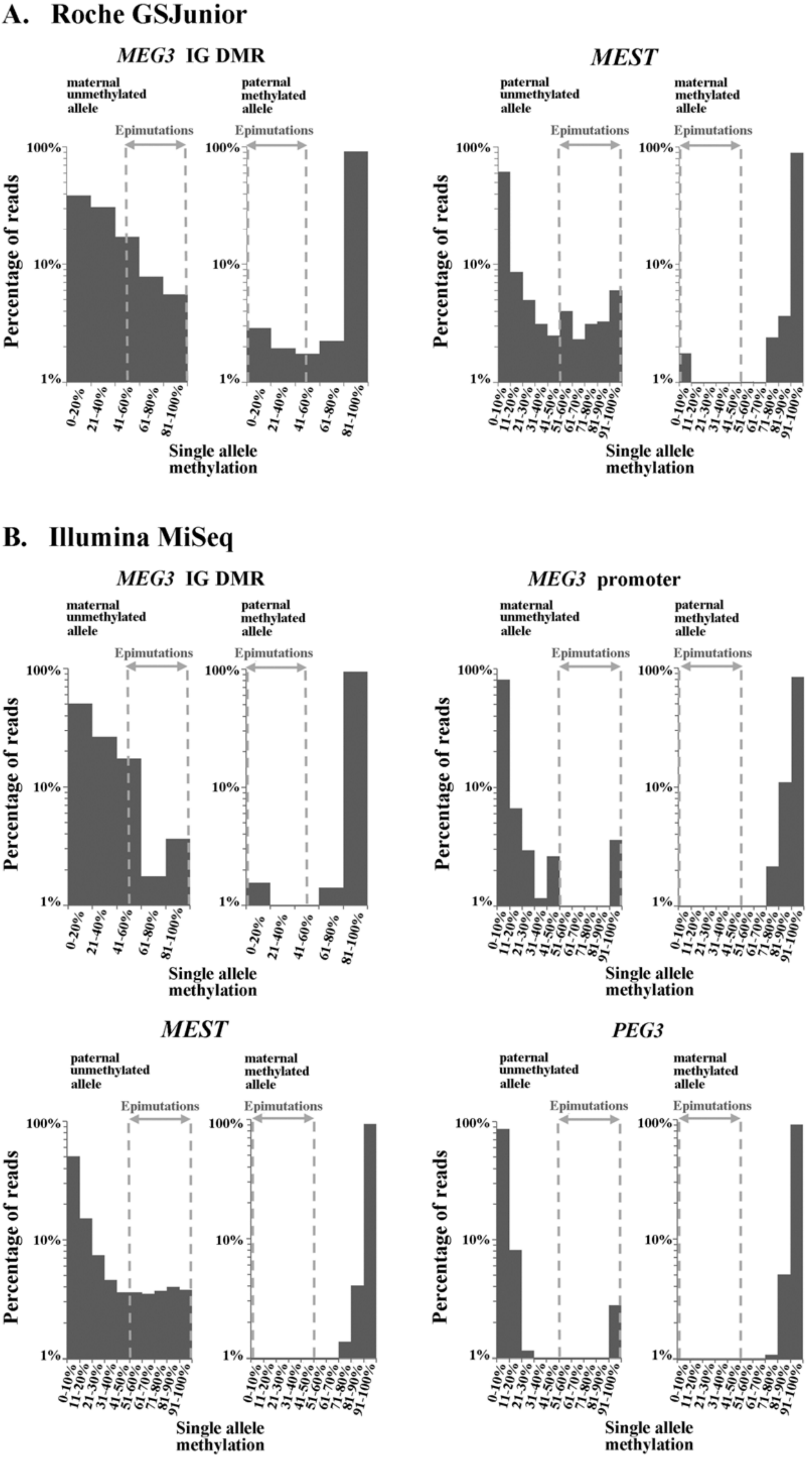
A. Roche GSJunior
MEG3 IG DMR
maternal unmethylated allele
paternal methylated allele
MEST
paternal unmethylated allele
maternal methylated allele
Epimutations
Percentage of reads
100%
10%
1%
0-20%
21-40%
41-60%
61-80%
81-100%
0-10%
11-20%
21-30%
31-40%
41-50%
51-60%
61-70%
71-80%
81-90%
91-100%
Single allele methylation
B. Illumina MiSeq
MEG3 IG DMR
MEG3 promoter
MEST
PEG3

S1 Fig. Methylation percentage distribution of non-imprinted (unmethylated) and imprinted (methylated) alleles. A) Methylation levels of paternal and maternal alleles of the paternally imprinted *MEG3* IG DMR and the maternally imprinted *MEST*, measured by DBS with the Roche GSJunior. B) Methylation percentage distribution of the paternally imprinted *MEG3* IG DMR and *MEG3* promoter as well as the maternally imprinted *MEST* and *PEG3* DMRs, measured by DBS with the Illumina MiSeq. Epimutations indicate alleles with >50% aberrantly (de)methylated CpGs.
