# Supplementary material for: Hypermethylation of the non-imprinted maternal MEG3 and paternal MEST alleles is highly variable among normal individuals
Source: PLoS One. 2017 Aug 30;12(8):e0184030. doi: 10.1371/journal.pone.0184030 (PMC5576652; doi:10.1371/journal.pone.0184030)
Supplement: S2 Fig — (PDF) [file pone.0184030.s002.pdf]

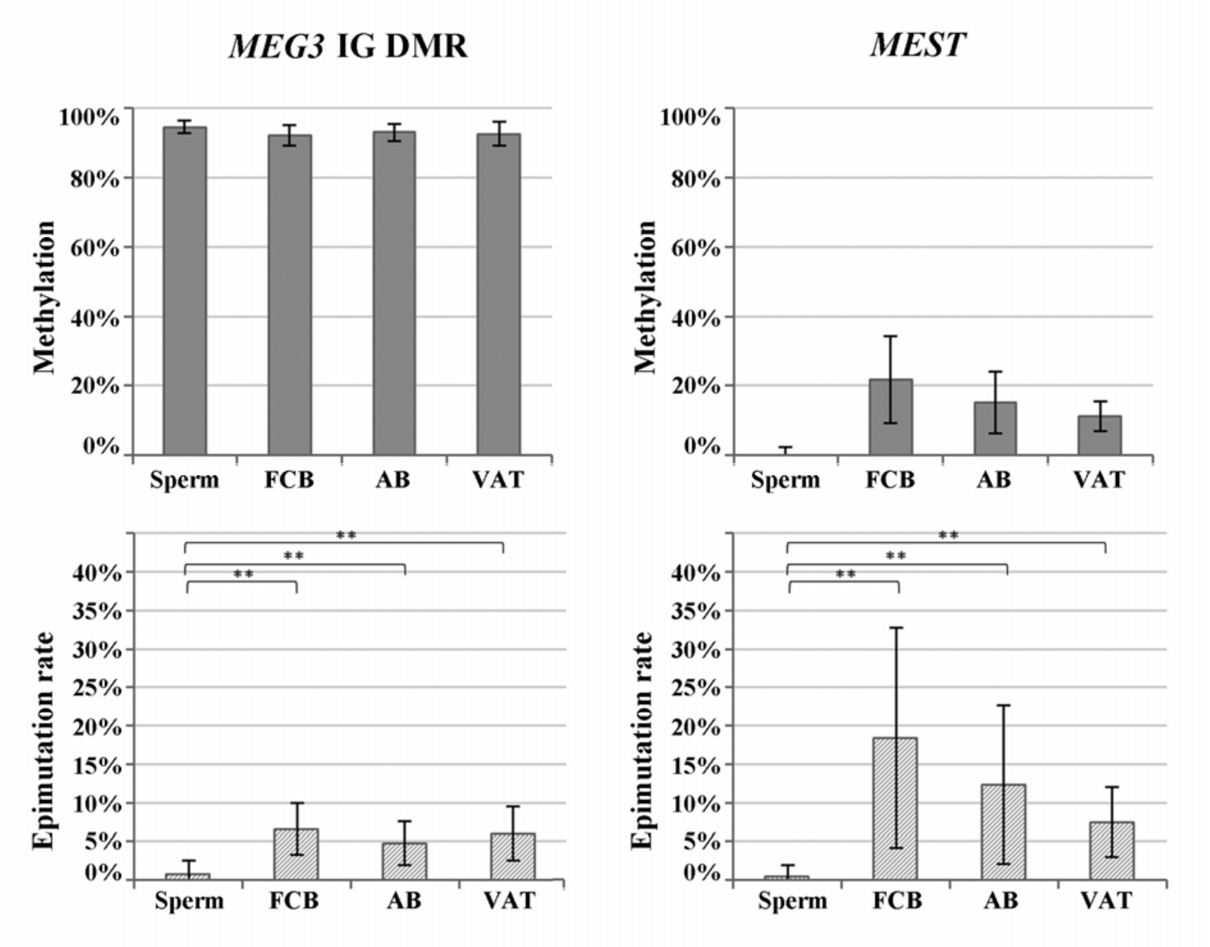

S2 Fig. Mean methylation and epimutation rates of the paternal *MEG3* IG DMR and the *MEST* allele in sperm, compared to FCB, AB and VAT. Please note that the epimutation rate in sperm, in particular of the unmethylated sperm *MEST* allele is significantly lower than in somatic tissues.
