# Supplementary material for: Hypermethylation of the non-imprinted maternal MEG3 and paternal MEST alleles is highly variable among normal individuals
Source: PLoS One. 2017 Aug 30;12(8):e0184030. doi: 10.1371/journal.pone.0184030 (PMC5576652; doi:10.1371/journal.pone.0184030)
Supplement: S3 Fig — (PDF) [file pone.0184030.s003.pdf]

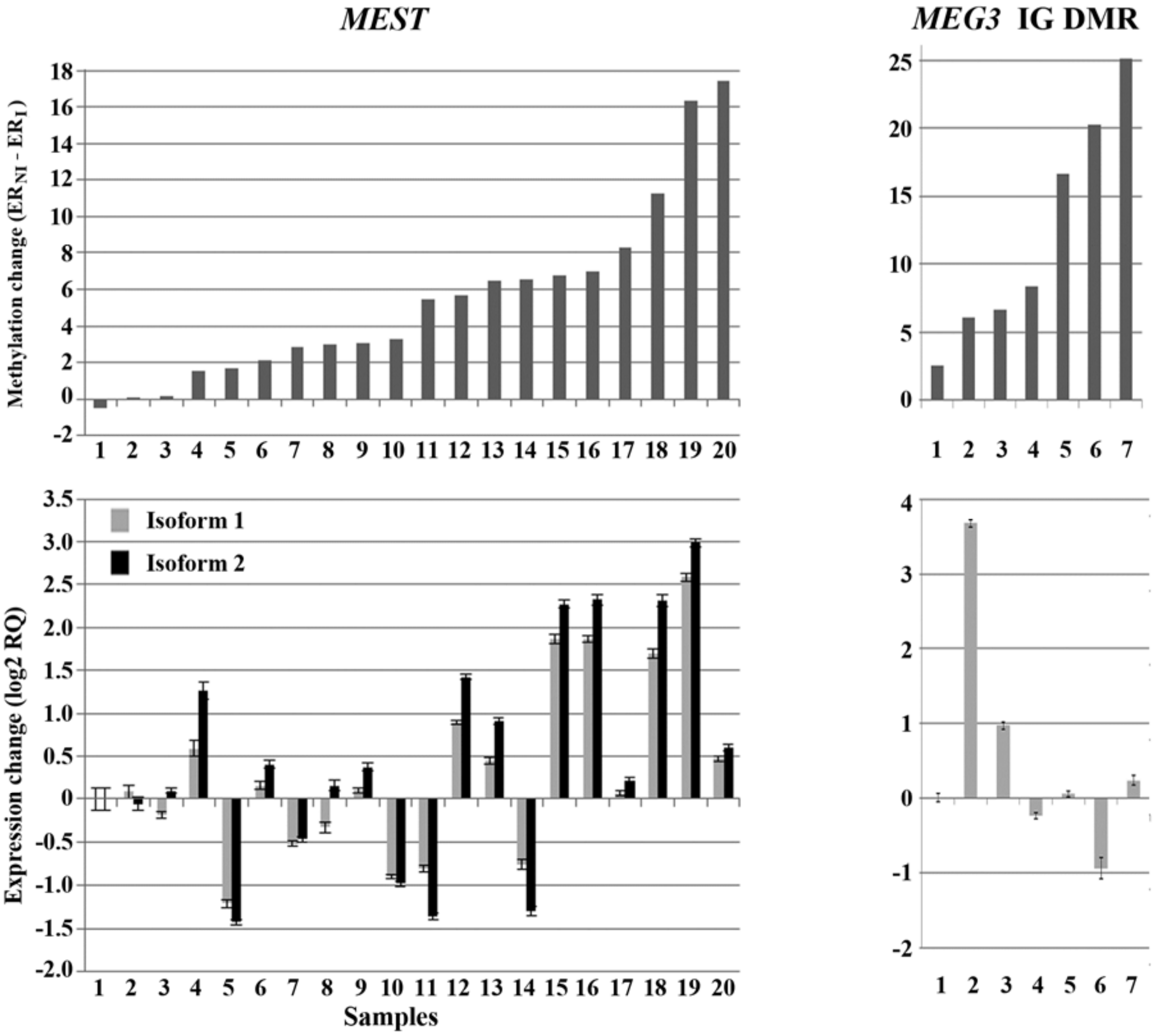

S3 Fig. Relationship between methylation and expression of *MEST* and *MEG3* IG DMR in VAT samples. The upper panels indicate the hypermethylation level of the analyzed samples. The difference between ERs on the non-imprinted and the imprinted allele (ER_{NI}-ER_I) is used as a measure of HNA. Sample 1 shows the lowest, sample 20 and 7, respectively, the highest methylation level. The lower panels present the expression levels of *MEST* (assays 1 and 2) and *MEG3* in the same samples, determined by RT-qPCR. A log2 RQ values of 1 correspond to an expression doubling and of -1 to a division in half (compared to sample 1).
