# Supplementary material for: Hypermethylation of the non-imprinted maternal MEG3 and paternal MEST alleles is highly variable among normal individuals
Source: PLoS One. 2017 Aug 30;12(8):e0184030. doi: 10.1371/journal.pone.0184030 (PMC5576652; doi:10.1371/journal.pone.0184030)
Supplement: S1 Table — (PDF) [file pone.0184030.s004.pdf]

S1 Table. Pyrosequencing primers for genotyping.

Gene	Primer	Sequence (5'-3')	Amplicon length (bp)	Chromosomal location[a]	SNP
MEG3 IG DMR	Forward[b]	CCTTGGACAAGAGAGCACATAGT	174	Chr14: 100,809,314-100,809,488	rs7159412 (0.13 C/ 0.87 T)
	Reverse	GGTGAATCACAGGGAATGATG			
	Sequencing	CTGGCAAACCCAGTT			
MEG3 promoter (ENSR00000099925)	Forward	TGGTGGTCGCTCGAAAATC	164	Chr14: 100,827,114-100,827,278	rs10134980 (0.17 A/ 0.83 C)
	Reverse[b]	AGAAACCTGCCACGTAGCC			
	Sequencing	TTGCCATTTCCTTTGT			
MEST promoter (ENSR00000069136)	Forward[b]	CTCTGGTGCGACTTAAAGGATAG	120	Chr7: 130,492,915-130,493,035	rs2301335 (0.46 G/ 0.54 A)
	Reverse	TCGGCACTGCGATTATCC			
	Sequencing	AGGTGCGCCACGGAG			
PEG3 promoter (ENSR00000349150)	Forward	CTAGCGCACCCTCATGGC	90	Chr19: 56,840,621-56,840,711	rs2302376 (0.20 T/ 0.80 C)
	Reverse[b]	GCTGCGGGAGGAGAGGTTT			
	Sequencing	CCCAGGGTGGACATC			

[a] Ensemble release 85.
[b] Biotinylated at the 5' end.
