# Supplementary material for: Hypermethylation of the non-imprinted maternal MEG3 and paternal MEST alleles is highly variable among normal individuals
Source: PLoS One. 2017 Aug 30;12(8):e0184030. doi: 10.1371/journal.pone.0184030 (PMC5576652; doi:10.1371/journal.pone.0184030)
Supplement: S2 Table — (PDF) [file pone.0184030.s005.pdf]

S2 Table. Primers for deep bisulfite sequencing.

**Roche GSJunior**					
**Gene**	**Primer**	**Template-specific sequence (5'-3')**	**Amplicon length (bp)**	**Chromosomal location**[a]	**Number of CpGs**
MEG3 IG DMR	Forward	[b]GGGTGTAAAAAGTTATTTGGGATTAG	312	Chr14: 100,809,296-100,809,608	8 (5 analyzed)
	Reverse	[c]ACCAAAATACTAACTACTCCTTAAACA			
MEST promoter	Forward	[b]GATTTAAAGGATAGGTTTTAGTAT	296	Chr7: 130,492,924-130,493,220	22
	Reverse	[c]AACCAAAATAAACAATCCCTAC			
**Illumina MiSeq**					
**Gene**	**Primer**	**Sequence (5'-3')**	**Amplicon length (bp)**	**Chromosomal location**[a]	**Number of CpGs**
MEG3 IG DMR	Forward	AAGTGTGGTGAATTATAGGGAATGA	398	Chr14: 100,809,096-100,809,494	8 (5 analyzed)
	Reverse	TTTCAAATAAAACTTCCCACTCCTCTTAC			
MEG3 promoter	Forward	TTAGGAAATTTTTTAGAAATTTGTTA	390	Chr14: 100,826,902-100,827,292	17
	Reverse	ATAACCCCCTAAAATCAATCCTTAC			
MEST promoter	Forward	AGTTATTGTTGTAGTATGATTTAGGATTTT	476	Chr7: 130,492,721-130,493,197	33
	Reverse	CAACATAACAATTTAATCACATC			
PEG3 promoter	Forward	TTTTGGGGGTTGTATTTTTATTATTTAATT	408	Chr19: 56,840,575-56,840,983	26
	Reverse	ACTCACCTCACCTCAATACTAC			

[a] Ensemble release 85.
[b] upstream universal adapter 1: CTTGCTTCCTGGCACGAG
[c] upstream universal adapter 2: CAGGAAACAGCTATGAC
