# Supplementary material for: Hypermethylation of the non-imprinted maternal MEG3 and paternal MEST alleles is highly variable among normal individuals
Source: PLoS One. 2017 Aug 30;12(8):e0184030. doi: 10.1371/journal.pone.0184030 (PMC5576652; doi:10.1371/journal.pone.0184030)
Supplement: S3 Table — (PDF) [file pone.0184030.s006.pdf]

S3 Table. Primers for reverse transcription quantitative real-time PCR.

**Reference Genes**				
	**Primer**	**Template-specific sequence (5'-3')**	**Amplicon length (bp)**	**Transcript**
GAPDH	Forward	TGCACCACCAACTGCTTAGC	87	GAPDH-001 ENST00000229239 (exon 7/8)
	Reverse	GGCATGGACTGTGGTCATGAG		
HPRT1	Forward	TGACACTGGCAAAACAATGCA	94	HPRT1-201 ENST00000298556.7 (exon 6/7)
	Reverse	GGTCCTTTTCACCAGCAAGCT		
IPO8	Forward	CGAGCTAGATCTTGCTGGGT	87	IPO8-201 ENST00000256079.8 (exons 13/14)
	Reverse	CGCTAATTCAACGGCATTTCTT		
RPLP0	Forward	GAACACCATGATGCGCAAGG	80	RPLP0-202 ENST00000313104.9 (exons 3/4)
	Reverse	CCCGGATATGAGGCAGCA		
**Genes of Interest**				
MEG3	Forward	GGCCTCTCGTCTCCTTCCT	80	MEG3-025 ENST00000556736 (exons 6/7)
	Reverse	GGGTCCCACATTCGAGGTC		
MEST assay 1	Forward	ATGAGGGAGTGGTGGGTCC	88	MEST-001 ENST00000223215.4 (exon 2)
	Reverse	CAGGGGAGAGCTGAGGGG		
MEST assay 2	Forward	GTCCTGTAGGCAAGGTCTTACC	90	MEST-002 ENST00000341441.5 (exons 1/2)
	Reverse	AGGTACGCAGCAAGCAGG		
