# Supplementary material for: Hypermethylation of the non-imprinted maternal MEG3 and paternal MEST alleles is highly variable among normal individuals
Source: PLoS One. 2017 Aug 30;12(8):e0184030. doi: 10.1371/journal.pone.0184030 (PMC5576652; doi:10.1371/journal.pone.0184030)
Supplement: S4 Table — (PDF) [file pone.0184030.s007.pdf]

S4 Table. DBS and expression measurements of all analyzed samples.

			Methylation									Expression	
			Maternal allele			Paternal allele			Both alleles				
**Gene**	**Tissue (ID)**	**Gender**	**SNP**	**Mean methylation**	**Epimutation rate**	**SNP**	**Mean methylation**	**Epimutation rate**	**SNP ratio**	**Reads**	**Mean methylation**	**ΔΔCt value**	**ΔCt SE**
**Roche GSJunior**													
MEG3 IG-DMR	VAT 1	female	G	36.0	20.7	A	95.3	4.0	0.84	866	68.2	-0.050	0.045
	VAT 2	female	A	52.0	52.1	G	96.0	2.8	0.72	767	70.4		
	VAT 3	female	A	29.6	25.4	G	91.4	5.1	0.56	762	51.6	0.939	0.133
	VAT 4	male	G	35.1	30.3	A	93.1	5.2	0.99	308	63.9		
	VAT 5	male	A	33.2	30.0	G	93.5	6.0	0.4	290	50.5		
	VAT 6	male	G	19.3	7.8	A	94.5	5.2	0.85	779	59.8	0.000	0.060
	VAT 7	female	G	20.1	12.4	A	94.0	4.3	0.87	1041	59.7		
	VAT 8	female	G	34.3	28.4	A	95.0	3.2	0.81	849	67.8	-0.235	0.061
	VAT 9	male	G	23.9	17.5	A	86.9	11.4	0.94	992	56.4	-3.673	0.053
	VAT 10	female	A	30.5	21.9	G	91.6	8.1	0.71	774	55.8		
	VAT 11	female	G	29.8	21.4	A	84.3	14.7	0.81	812	59.9	-0.967	0.044
	VAT 12	female	A	19.8	12.3	G	94.5	3.9	0.78	999	52.5	0.244	0.036
	VAT 13	male	A	20.0	14.9	G	95.4	3.4	0.75	832	52.4		
	AB 1	female	G	28.5	20.1	A	94.3	4.6	0.84	916	64.1		
	AB 2	female	A	33.0	28.2	G	84.0	15.2	0.76	838	55.2		
	AB 3	female	A	28.1	21.0	G	91.9	7.0	0.68	781	54.0		
	AB 4	male	A	25.6	17.2	G	95.3	3.1	0.58	696	51.1		
	AB 5	male	G	27.1	14.6	A	94.8	4.4	0.85	671	63.7		
	AB 6	female	G	24.3	17.7	A	95.0	3.7	0.68	867	66.4		
	AB 7	female	G	32.3	25.5	A	94.9	4.6	0.77	883	67.6		
	AB 8	male	G	21.4	14.3	A	91.9	7.7	0.76	1070	61.5		
	AB 9	male	A	26.1	14.7	G	94.2	3.7	0.68	730	53.8		
	AB 10	female	A	26.5	21.2	G	93.2	6.2	0.74	764	54.7		
	AB 11	female	G	18.8	10.0	A	94.4	3.6	0.72	1006	62.8		
	AB 12	female	A	25.6	20.9	G	95.1	2.1	0.63	871	52.4		
	AB 13	male	A	30.8	22.7	G	92.7	6.3	0.55	540	53.0		
	AB 14	male	A	44.7	43.8	G	90.6	7.1	0.65	607	62.8		

	AB 15	female	G	59.5	65.5	A	93.6	3.2	0.76	764	79.1		
	AB 16	male	A	21.7	16.3	G	95.4	3.2	0.76	646	53.6		
	AB 17	male	A	34.4	27.7	G	95.1	2.1	0.96	826	65.4		
	AB 18	female	A	31.4	27.9	G	93.3	1.9	0.49	165	51.6		
	AB 19	female	G	30.3	20.7	A	91.3	7.0	0.96	474	61.4		
	AB 20	female	A	35.1	27.4	G	93.4	5.0	0.65	714	58.0		
	AB 21	male	A	26.0	16.0	G	91.7	6.8	0.71	748	53.3		
	AB 22	male	G	36.5	30.0	A	92.3	5.7	0.8	1262	67.4		
	AB 23	male	G	23.3	13.2	A	92.9	6.5	0.92	650	59.6		
	FCB 1	female	A	20.9	9.7	G	95.0	2.0	0.92	731	56.4		
	FCB 2	male	G	19.9	10.9	A	92.4	6.3	0.85	1022	59.0		
	FCB 3	female	G	18.9	13.7	A	89.8	8.8	0.98	998	54.6		
	FCB 4	female	G	24.7	14.9	A	87.3	12.6	0.87	1011	58.1		
	FCB 5	male	A	22.3	7.0	G	93.4	5.0	0.45	766	44.4		
	FCB 6	female	A	20.7	5.6	G	96.8	2.6	0.79	958	54.3		
	FCB 7	female	A	22.4	12.2	G	94.1	5.1	0.69	966	51.8		
	FCB 8	female	G	16.9	4.6	A	89.1	9.7	0.98	1227	53.4		
	FCB 9	female	G	19.4	9.3	A	89.4	9.8	0.62	817	62.9		
	FCB 10	female	G	22.9	13.1	A	93.1	6.3	0.98	483	57.6		
	FCB 11	female	A	22.9	13.6	G	91.3	9.0	0.76	181	52.4		
	FCB 12	female	A	24.0	20.5	G	94.8	3.7	0.87	576	56.9		
	FCB 13	male	A	26.5	17.9	G	90.0	9.8	0.56	482	49.1		
	FCB 14	male	G	26.6	16.7	A	86.8	11.8	0.86	869	58.9		
	FCB 15	female	A	25.4	14.8	G	87.4	12.1	0.84	1233	53.7		
	FCB 16	male	A	23.6	11.5	G	94.6	3.0	0.75	547	53.9		
	FCB 17	female	G	18.8	10.8	A	98.0	1.1	0.71	157	65.2		
	FCB 18	male	G	23.4	16.7	A	86.7	12.8	0.98	962	54.8		
	FCB 19	male	A	23.3	12.0	G	95.0	3.7	0.6	931	50.1		
	FCB 20	female	G	20.7	9.2	A	93.0	6.0	0.97	949	57.4		
	FCB 21	male	A	29.1	20.2	G	94.3	4.4	0.56	960	52.5		
	FCB 22	female	G	29.6	22.1	A	92.8	5.0	0.85	1380	63.8		
	FCB 23	female	A	22.1	13.7	G	95.5	4.1	0.88	1044	56.1		
	FCB 24	female	A	22.1	12.1	G	93.3	5.2	0.69	947	51.6		
	FCB 25	male	A	24.1	15.6	G	95.9	2.4	0.72	814	54.2		
	FCB 26	male	G	24.9	20.4	A	93.3	5.6	0.97	568	59.5		
	FCB 27	male	G	21.3	10.7	A	91.8	6.4	0.90	650	58.4		
	FCB 28	male	A	23.2	12.3	G	89.9	8.7	0.64	294	49.5		

	FCB 29	male	G	24.2	14.4	A	90.3	8.7	0.78	1065	61.3		
	FCB 30	male	A	25.8	17.0	G	92.4	6.8	0.81	459	55.9		
MEST promoter	VAT 1	male	G	97.3	1.1	A	11.0	7.9	0.74	638	47.7	-2.273	0.051
	VAT 2	female	A	95.6	2.3	G	10.5	6.1	0.83	754	49.1		
	VAT 3	female	A	95.4	1.6	G	8.7	4.5	0.96	649	51.1	-0.154	0.063
	VAT 4	female	G	96.9	1.3	A	21.0	17.7	0.88	570	61.5	-2.985	0.053
	VAT 5	male	A	95.4	1.9	G	10.7	8.4	0.77	738	47.5		
	VAT 6	female	A	93.4	3.1	G	6.1	3.2	0.64	1070	40.2	0.061	0.075
	VAT 7	female	A	94.6	3.2	G	13.5	9.8	0.87	862	51.2	1.292	0.055
	VAT 8	male	A	94.9	3.6	G	7.1	3.1	0.81	933	46.5	0.000	0.130
	VAT 9	male	G	95.8	2.2	A	11.4	7.6	0.88	780	50.8	1.359	0.034
	VAT 10	male	G	96.5	1.5	A	13.6	9.8	0.91	513	56.9	-0.210	0.035
	VAT 11	male	A	95.3	2.5	G	5.2	2.7	0.86	696	46.9	-0.098	0.035
	VAT 12	female	A	95.5	2.4	G	7.8	4.1	0.79	658	46.6	1.424	0.032
	VAT 13	female	A	94.8	2.3	G	12.6	8.0	0.92	919	52.1	-1.426	0.037
	VAT 14	male	G	94.1	2.8	A	10.8	6.1	0.92	681	54.2	0.979	0.029
	VAT 15	male	A	94.5	2.4	G	8.1	5.2	0.86	820	48.0	0.473	0.033
	VAT 16	male	A	95.6	2.4	G	9.4	5.7	0.92	913	50.8		
	VAT 17	male	A	98.1	0.3	G	5.8	1.9	0.90	616	49.5	-1.269	0.098
	VAT 18	female	G	96.2	1.0	A	10.2	8.0	0.84	713	56.9	-2.322	0.060
	VAT 19	male	G	96.1	1.8	A	8.4	3.9	0.95	705	51.1	-0.404	0.051
	VAT 20	female	G	94.9	1.8	A	15.6	13.0	0.69	581	62.5	-2.317	0.069
	VAT 21	male	G	95.7	2.0	A	12.8	10.7	0.97	685	54.8		
	VAT 22	female	G	94.2	3.3	A	14.5	9.8	0.90	894	52.2	-0.912	0.037
	VAT 23	female	G	96.4	1.3	A	8.2	4.4	0.77	887	46.5	-0.375	0.047
	VAT 24	male	G	95.3	2.3	A	22.7	19.8	0.65	939	51.3	-0.593	0.039
	AB 1	female	A	94.9	2.4	G	9.6	7.1	0.84	1392	48.5		
	AB 2	male	A	94.9	2.2	G	14.0	11.8	0.73	1180	60.7		
	AB 3	female	G	93.5	3.7	A	18.3	15.4	0.87	1207	53.3		
	AB 4	female	A	93.3	4.5	G	7.7	5.4	0.62	1507	40.3		
	AB 5	male	G	90.2	5.8	A	50.3	57.6	0.94	1473	70.8		
	AB 6	male	A	94.1	3.7	G	16.3	11.6	0.95	850	56.2		
	AB 7	female	A	94.5	2.7	G	8.4	5.1	0.82	1090	55.7		
	AB 8	male	G	92.7	5.3	A	8.2	5.3	0.36	1419	30.8		
	AB 9	male	A	92.5	4.5	G	6.4	3.8	0.44	1455	32.7		
	AB 10	female	G	94.4	2.0	A	17.2	16.7	0.49	1512	42.7		
	AB 11	female	A	94.8	2.7	G	20.8	19.4	0.85	1318	60.9		

	AB 12	male	A	95.7	1.6	G	10.3	6.7	0.87	1084	50.0		
	AB 13	female	A	95.4	1.4	G	22.2	19.7	0.92	1243	57.1		
	AB 14	male	G	95.1	2.1	A	14.2	11.5	0.89	1032	52.2		
	AB 15	female	A	94.6	2.9	G	14.2	11.6	0.87	1754	57.3		
	AB 16	male	G	94.3	2.5	A	9.1	7.1	0.65	1349	42.5		
	AB 17	male	G	95.8	1.9	A	35.5	34.4	0.78	1138	69.3		
	AB 18	male	A	94.7	2.6	G	11.2	8.8	0.88	1636	55.6		
	AB 19	female	A	92.6	5.5	G	5.9	3.6	0.52	1451	35.4		
	AB 20	male	G	94.8	2.0	A	10.1	7.6	0.68	1508	44.4		
	AB 21	male	G	96.6	1.8	A	17.0	16.3	0.84	1214	53.4		
	AB 22	female	A	94.9	2.3	G	11.3	8.6	0.85	1425	49.7		
	AB 23	male	A	93.3	4.3	G	9.8	6.7	0.82	1201	47.4		
	AB 24	female	G	96.4	1.4	A	15.7	11.8	0.90	1495	53.9		
	AB 25	female	A	93.7	4.0	G	21.6	20.2	0.98	839	57.9		
	AB 26	male	G	90.9	9.1	A	19.9	20.0	0.44	72	41.6		
	AB 27	male	G	95.4	1.7	A	14.2	12.7	0.84	1380	51.3		
	AB 28	female	A	96.2	1.5	G	16.0	12.9	0.95	1092	55.1		
	AB 29	female	G	94.4	1.8	A	13.0	9.7	0.90	825	51.5		
	AB 30	male	A	92.1	3.8	G	11.1	9.1	0.60	1043	41.6		
	AB 31	female	G	92.7	2.6	A	29.4	24.8	0.92	240	59.7		
	AB 32	female	A	93.0	3.2	G	18.9	6.8	0.83	1327	52.6		
	AB 33	female	A	91.6	4.4	G	9.8	6.0	0.45	1522	32.9		
	AB 34	female	A	90.9	4.8	G	7.5	4.0	0.55	502	28.4		
	AB 35	female	A	91.4	4.9	G	10.8	6.3	0.55	1033	39.4		
	AB 36	female	A	88.7	5.6	G	7.0	2.3	0.20	1279	27.0		
	FCB 1	female	G	95.9	0.9	A	26.1	22.7	0.64	826	53.5		
	FCB 2	female	G	95.0	0.7	A	37.8	38.8	0.89	1055	68.1		
	FCB 3	female	A	97.8	0.6	G	16.5	12.6	0.85	1048	60.1		
	FCB 4	female	A	97.2	0.0	G	14.5	11.1	0.92	692	57.3		
	FCB 5	female	A	97.4	0.7	G	26.2	25.9	0.96	835	62.5		
	FCB 6	female	G	96.1	0.9	A	41.2	43.2	0.67	973	74.0		
	FCB 7	female	A	96.2	1.7	G	15.6	11.6	0.70	1089	62.9		
	FCB 8	female	A	96.1	0.8	G	16.8	11.7	0.75	677	62.1		
	FCB 9	male	A	96.2	1.4	G	29.0	15.7	0.95	1016	57.6		
	FCB 10	male	G	94.2	2.9	A	16.3	12.0	0.94	999	56.3		
	FCB 11	male	G	95.0	1.2	A	28.8	25.9	0.86	945	64.5		
	FCB 12	male	G	81.5	14.6	A	38.3	37.4	0.94	1056	65.0		

	FCB 13	male	G	92.8	5.3	A	12.7	7.4	0.76	480	47.4		
	FCB 14	female	G	89.3	6.3	A	18.8	14.7	0.62	703	45.7		
	FCB 15	male	G	96.2	1.4	A	23.6	18.7	0.96	1198	59.2		
	FCB 16	female	G	93.4	2.6	A	17.3	12.5	0.71	644	48.9		
	FCB 17	male	A	87.7	9.4	G	24.0	14.8	0.60	1613	45.7		
	FCB 18	male	A	97.0	0.9	G	12.5	4.3	0.86	1223	51.5		
	FCB 19	female	G	94.0	2.9	A	28.5	24.3	0.88	1946	59.2		
	FCB 20	male	G	98.7	0.2	A	10.9	10.5	0.39	782	73.4		
	FCB 21	male	G	94.9	2.5	A	28.8	27.9	0.44	1246	55.3		
	FCB 22	female	G	92.2	4.3	A	14.0	11.6	0.38	1172	35.7		
	FCB 23	female	A	81.9	15.4	G	8.6	4.9	0.32	1137	26.2		
	FCB 24	female	G	85.8	10.1	A	8.9	3.5	0.15	1048	19.1		
	FCB 25	female	A	94.0	3.8	G	6.3	3.1	0.35	1311	29.1		
	FCB 26	female	G	91.3	4.4	A	10.2	5.7	0.12	835	18.9		
	FCB 27	female	G	87.9	8.3	A	8.8	4.0	0.12	1281	17.0		
	FCB 28	female	A	83.3	12.6	G	10.0	5.1	0.19	2067	21.5		
	FCB 29	male	A	95.6	1.4	G	6.5	2.1	0.43	1222	33.3		
	FCB 30	female	A	93.2	2.6	G	13.4	7.6	0.43	1421	37.2		
	FCB 31	male	G	91.9	4.5	A	21.3	16.8	0.36	1083	40.0		
	FCB 32	female	A	95.5	2.0	G	12.7	11.0	0.69	990	38.6		
	FCB 33	female	G	94.9	1.1	A	44.5	44.9	0.71	1002	66.9		
	FCB 34	female	G	96.6	0.0	A	22.2	13.6	0.55	68	48.5		
	FCB 35	male	G	96.6	0.8	A	42.2	43.0	0.83	880	71.9		
	FCB 36	male	G	86.1	12.9	A	58.5	61.5	0.85	886	73.4		
	FCB 37	male	A	97.6	0.8	G	10.8	7.0	0.64	1863	63.9		
	FCB 38	male	A	97.8	0.7	G	25.6	23.7	0.81	611	57.9		
	FCB 39	female	A	97.7	0.3	G	9.3	5.2	0.87	1163	56.6		
	FCB 40	female	G	96.0	1.0	A	21.7	17.3	0.87	1304	61.4		
	FCB 41	female	G	96.3	0.8	A	37.0	36.7	0.94	1002	67.6		
	FCB 42	female	A	95.8	1.6	G	16.8	12.2	1.00	1390	56.2		
	FCB 43	female	G	96.6	0.2	A	33.5	29.7	0.88	805	67.0		
	FCB 44	female	G	96.4	0.6	A	44.5	46.4	0.89	751	69.0		
	FCB 45	male	A	85.9	12.2	G	33.2	33.4	0.69	1049	54.6		
	FCB 46	female	A	97.1	0.6	G	29.0	28.6	0.53	1115	73.6		
	FCB 47	female	A	89.9	6.6	G	5.4	2.4	0.11	1106	13.5		
	FCB 48	male	A	84.4	12.8	G	5.4	2.8	0.42	1045	31.9		
	FCB 49	male	A	92.0	6.4	G	7.6	4.5	0.46	979	44.5		

	FCB 50	female	G	94.6	1.5	A	31.5	31.5	0.64	988	56.0		
**Illumina MiSeq**													
MEG3	FCB 1	female	G	19.3	14.1	A	92.2	6.3	0.63	96542	47.4		
IG-DMR	FCB 2	female	G	25.9	25.9	A	93.5	6.6	0.76	57108	64.3		
	FCB 3	female	G	21.1	14.0	A	94.7	4.2	0.72	68561	52.0		
	FCB 4	female	G	28.2	24.5	A	94.9	1.4	0.80	46030	57.8		
	FCB 5	male	A	21.3	14.2	G	95.4	3.5	0.75	83951	53.2		
	FCB 6	male	G	25.2	20.6	A	96.1	2.3	0.48	42934	48.2		
	FCB 7	female	A	19.8	8.7	G	96.2	2.6	0.83	63640	54.6		
	FCB 8	female	G	17.7	10.7	A	96.3	2.9	0.73	61522	50.8		
	FCB 9	female	A	21.2	11.6	G	96.3	2.5	0.88	66298	56.4		
	FCB 10	male	G	13.4	4.9	A	96.3	2.4	0.47	44799	39.8		
	FCB 11	female	A	20.0	8.2	G	96.4	1.2	0.82	82505	54.4		
	FCB 12	female	G	17.6	12.3	A	96.5	2.6	0.70	46251	50.0		
	FCB 13	male	A	21.1	11.5	G	96.9	1.6	0.79	106241	54.6		
	FCB 14	female	A	23.0	13.4	G	97.0	1.6	0.87	108017	57.4		
	FCB 15	female	A	24.0	14.8	G	97.1	1.3	0.68	126975	53.6		
	FCB 16	male	G	21.8	15.5	A	97.1	1.3	0.64	91889	51.2		
	FCB 17	female	A	22.9	12.6	G	97.4	1.1	0.88	56532	57.8		
	FCB 18	male	A	20.1	9.1	G	97.4	1.2	0.58	45617	48.4		
	FCB 19	male	A	25.8	15.4	G	97.6	1.0	0.76	70929	56.9		
	FCB 20	female	A	26.0	16.0	G	97.8	0.9	0.90	81195	59.9		
	FCB 21	female	G	21.6	14.6	A	87.9	11.3	0.80	37529	51.0		
	FCB 22	female	G	22.3	18.3	A	91.6	7.0	0.63	21538	49.2		
	FCB 23	female	A	17.2	7.6	G	92.7	7.1	0.77	34932	50.0		
	FCB 24	female	A	16.9	8.0	G	93.1	4.5	0.69	21096	47.9		
	FCB 25	male	G	20.1	16.2	A	93.5	4.5	0.65	35427	49.0		
	FCB 26	female	A	17.2	9.3	G	93.6	3.6	0.87	38009	52.8		
	FCB 27	female	G	24.3	17.8	A	93.9	6.2	0.52	37675	48.1		
	FCB 28	male	G	20.7	16.8	A	94.8	3.8	0.62	24866	49.0		
	FCB 29	female	A	24.4	13.1	G	95.0	4.7	0.56	29650	49.9		
	FCB 30	female	G	19.7	15.8	A	95.3	3.3	0.72	35269	51.4		
	FCB 31	male	A	17.5	8.8	G	95.8	1.1	0.75	26799	51.0		
	FCB 32	female	A	22.3	11.6	G	96.0	2.5	0.79	25557	54.8		
	FCB 33	female	G	17.4	9.2	A	96.0	1.3	0.62	20817	47.6		
	FCB 34	male	A	18.7	9.8	G	96.0	2.7	0.82	31476	53.5		
	FCB 35	male	A	20.7	13.6	G	96.2	3.8	0.84	31939	55.2		

	FCB 36	male	A	21.9	13.5	G	96.5	2.5	0.49	31832	46.5		
	FCB 37	female	A	27.2	18.8	G	96.8	1.7	0.74	25967	56.7		
	FCB 38	female	G	22.6	19.0	A	96.9	1.5	0.62	33211	51.0		
	FCB 39	female	G	17.3	11.5	A	97.2	1.3	0.60	21382	47.3		
	FCB 40	female	A	23.0	11.5	G	97.9	1.1	0.76	25685	55.2		
	FCB 41	female	A	17.7	9.5	G	97.8	1.2	0.62	9160	48.5		
	FCB 42	male	A	21.6	9.4	G	96.5	2.4	0.62	17551	50.4		
	FCB 43	male	A	22.8	13.3	G	98.1	0.9	0.60	12625	51.0		
	FCB 44	male	G	21.0	17.8	A	96.1	2.9	0.73	19464	52.7		
	FCB 45	-	G	19.9	17.3	A	89.4	9.9	0.84	10607	51.6		
MEG3 promoter	FCB 1	female	T	3.1	1.8	G	91.8	0.2	0.42	10546	65.7		
	FCB 2	male	G	2.0	0.6	T	92.9	0.7	0.80	23786	52.6		
	FCB 3	female	G	18.5	5.7	T	93.8	0.6	0.93	19736	57.6		
	FCB 4	male	G	23.7	22.5	T	96.5	0.5	0.51	18289	72.0		
	FCB 5	female	G	6.4	0.9	T	95.8	0.8	0.65	11546	60.6		
	FCB 6	male	T	4.1	2.5	G	95.2	0.5	0.56	13563	62.3		
	FCB 7	male	G	23.8	22.6	T	97.0	0.5	0.51	12861	93.2		
	FCB 8	female	T	4.5	2.1	G	96.1	0.5	0.37	24920	71.6		
	FCB 9	male	G	6.4	2.5	T	95.5	1.1	0.44	15864	68.1		
	FCB 10	male	T	6.8	4.6	G	95.6	0.6	0.30	21692	75.0		
	FCB 11	male	T	16.0	2.6	G	93.7	1.2	0.99	20895	54.7		
	FCB 12	male	T	4.0	1.5	G	94.6	2.5	0.46	23011	66.1		
	FCB 13	female	G	54.8	54.5	T	93.2	3.9	0.92	19010	73.2		
	FCB 14	male	G	4.4	1.4	T	94.5	1.3	0.45	11282	66.7		
	FCB 15	female	G	11.5	8.4	T	95.9	0.6	0.42	16672	70.8		
	FCB 16	male	G	6.0	4.9	T	94.8	0.6	0.50	15240	65.2		
	FCB 17	female	T	5.4	3.0	G	95.2	0.7	0.42	14450	68.7		
	FCB 18	male	G	4.8	2.1	T	96.7	0.3	0.30	7814	75.3		
	FCB 19	female	T	20.7	3.7	G	95.9	0.5	0.59	9638	68.1		
	FCB 20	female	T	7.4	4.9	G	95.5	1.0	0.28	9677	76.4		
	FCB 21	female	T	6.2	4.1	G	95.2	0.3	0.27	7686	76.1		
	FCB 22	male	G	3.6	1.1	T	94.1	0.7	0.41	23966	68.0		
	FCB 23	male	G	4.9	2.7	T	93.6	1.0	0.71	4556	56.9		
	FCB 24	male	G	7.0	4.7	T	93.6	0.6	0.59	10011	61.6		
	FCB 25	male	G	4.2	0.5	T	94.9	1.2	0.91	7397	47.5		
	FCB 26	female	T	2.7	1.0	G	94.2	0.7	0.70	4187	56.6		
	FCB 27	male	T	4.5	2.7	G	92.4	2.7	0.46	6115	64.8		

	FCB 28	female	T	1.9	0.4	G	95.8	0.4	0.81	4915	53.8		
	FCB 29	male	G	2.8	0.3	T	97.2	0.1	0.90	6124	52.5		
	FCB 30	female	G	2.1	0.8	T	95.7	0.6	0.74	4845	55.9		
	FCB 31	female	T	4.7	1.8	G	92.3	3.8	0.64	6313	58.1		
MEST promoter	FCB 1	male	G	97.5	0.3	A	28.4	26.7	0.68	75600	56.4		
	FCB 2	female	G	96.1	0.7	A	23.1	16.2	0.94	33971	60.7		
	FCB 3	male	A	97.1	0.7	G	12.2	8.0	0.84	47917	50.8		
	FCB 4	male	A	95.6	3.4	G	13.2	12.4	0.91	31391	52.5		
	FCB 5	male	A	98.9	0.3	G	7.7	0.7	0.78	33682	59.0		
	FCB 6	male	A	93.7	4.6	G	14.2	10.5	0.80	48379	49.6		
	FCB 7	male	A	97.0	0.4	G	16.8	12.5	0.79	39400	52.4		
	FCB 8	female	A	98.0	0.3	G	12.6	5.9	0.73	38374	48.8		
	FCB 9	female	G	97.0	0.3	A	29.4	27.0	0.79	41692	59.6		
	FCB 10	female	A	96.0	0.7	G	14.9	9.5	0.87	21891	52.8		
	FCB 11	female	G	97.0	0.5	A	38.7	38.2	0.80	29498	64.7		
	FCB 12	female	G	96.5	0.5	A	31.5	27.7	0.80	38691	60.4		
	FCB 13	female	G	97.0	0.8	A	38.7	39.8	0.62	30379	61.0		
	FCB 14	male	G	93.1	4.6	A	42.1	41.7	0.53	25105	59.9		
	FCB 15	female	A	97.6	0.5	G	9.5	4.1	0.84	16491	49.9		
	FCB 16	female	G	97.6	0.5	A	31.3	29.7	0.84	19280	61.5		
	FCB 17	female	A	93.4	3.4	G	7.2	1.0	1.00	2139	50.4		
	FCB 18	female	G	96.3	0.8	A	19.7	13.6	0.81	3871	53.9		
	FCB 19	female	A	97.7	0.5	G	25.5	22.3	0.60	4144	52.6		
	FCB 20	female	A	97.2	0.5	G	20.7	16.3	0.73	4120	53.0		
	FCB 21	female	A	98.3	0.4	G	14.2	7.7	0.34	4343	35.8		
	FCB 22	female	G	97.6	0.7	A	26.9	23.7	0.33	2773	44.5		
	FCB 23	female	A	97.6	0.2	G	30.2	27.1	0.53	3240	53.7		
	FCB 24	male	G	95.5	1.8	A	11.7	5.6	0.81	3052	49.2		
	FCB 25	male	G	82.1	12.8	A	42.0	35.1	0.80	2028	59.8		
	FCB 26	female	A	96.4	1.2	G	19.5	14.1	0.64	12563	49.5		
	FCB 27	female	G	94.3	1.8	A	31.6	29.3	0.73	19344	58.1		
	FCB 28	male	G	87.5	12.7	A	28.4	29.5	0.76	14823	54.0		
	FCB 29	male	A	91.0	6.6	G	27.9	23.2	0.88	16439	61.4		
	FCB 30	female	G	94.7	2.4	A	22.4	14.9	0.93	70456	57.3		
	FCB 31	female	G	95.7	1.4	A	35.5	33.6	0.56	53291	57.1		
	FCB 32	female	A	97.8	0.5	G	21.3	16.9	0.73	51340	53.5		
	FCB 33	female	G	98.0	0.2	A	39.1	41.8	0.84	38763	66.0		

	FCB 34	male	A	97.3	0.3	G	28.9	27.1	0.59	68287	54.3		
	FCB 35	female	A	96.4	0.8	G	20.9	15.3	0.72	41703	52.4		
	FCB 36	male	G	97.6	1.1	A	17.8	10.9	0.92	34648	56.1		
	FCB 37	female	A	95.7	1.7	G	13.1	8.1	0.70	11908	47.1		
	FCB 38	female	A	97.5	0.9	G	24.3	22.2	0.72	12087	54.9		
	FCB 39	female	A	96.0	0.9	G	12.9	9.7	0.73	18955	48.1		
	FCB 40	male	G	94.2	2.2	A	26.7	21.9	0.71	12700	54.7		
	FCB 41	female	G	95.8	0.8	A	29.1	26.3	0.82	2494	59.1		
	FCB 42	male	A	95.9	1.7	G	11.4	5.8	0.74	3726	47.3		
	FCB 43	female	G	97.7	0.5	A	29.8	27.0	0.69	3174	57.5		
	FCB 44	female	G	96.9	1.1	A	33.0	32.4	0.57	2963	56.1		
	FCB 45	female	G	95.1	0.5	A	19.3	13.1	0.61	5870	48.0		
	FCB 46	male	A	97.5	1.2	G	7.2	3.7	0.63	3781	42.3		
	FCB 47		G	98.6	0.5	A	5.6	1.3	0.74	2336	45.1		
	FCB 48	female	G	96.1	0.7	A	21.1	21.0	0.72	3578	52.4		
	FCB 49		G	90.6	2.4	A	30.6	30.1	0.65	5171	54.2		
	FCB 50	female	A	95.7	0.9	G	12.6	9.0	0.38	2570	35.5		
	FCB 51	female	G	97.8	0.3	A	22.7	15.8	0.53	2283	48.8		
	FCB 52	female	G	95.7	0.8	A	26.4	21.6	0.54	6843	50.6		
	FCB 53	female	G	97.0	0.5	A	27.7	25.0	0.70	7745	56.3		
	FCB 54	male	A	93.6	1.7	G	13.1	9.7	0.85	5198	50.0		
	FCB 55	male	G	95.5	1.5	A	23.5	20.8	0.47	9667	46.6		
	FCB 56	male	A	97.6	0.3	G	13.4	7.9	0.36	3413	35.7		
	FCB 57	male	G	95.9	1.6	A	15.6	11.0	0.67	3406	47.8		
	FCB 58	female	G	84.8	13.6	A	20.0	18.9	0.90	2866	54.1		
PEG3 promoter	FCB 1	female	G	97.0	1.0	A	4.3	1.5	0.63	72914	61.1		
	FCB 2		A	93.6	0.8	G	3.6	0.9	0.48	59955	64.4		
	FCB 3	male	A	97.4	0.2	G	5.6	0.3	0.60	33754	63.0		
	FCB 4	male	A	94.3	1.9	G	3.9	0.4	0.80	56053	54.2		
	FCB 5	female	A	95.8	1.0	G	7.5	4.7	0.45	49570	68.4		
	FCB 6	male	G	93.4	3.7	A	2.7	0.6	0.60	39163	59.5		
	FCB 7	female	A	95.6	1.1	G	4.3	1.2	0.67	77415	58.8		
	FCB 8	female	A	98.0	0.3	G	19.4	17.3	0.95	43503	57.7		
	FCB 9	female	A	95.8	1.2	G	3.6	0.7	0.49	45740	65.6		
	FCB 10	male	A	95.0	1.1	G	3.1	0.6	0.58	38237	61.1		
	FCB 11	male	A	96.3	0.5	G	3.0	0.7	0.77	34305	55.8		
	FCB 12	male	G	94.0	5.4	A	15.6	14.0	0.72	42592	61.2		

	FCB 13	female	A	96.7	0.7	G	6.4	3.3	0.55	68966	64.7		
	FCB 14		G	92.2	0.6	A	9.8	0.6	0.67	12577	59.2		
	FCB 15	male	A	96.0	6.2	G	2.5	12.8	0.66	38934	58.7		
	FCB 16	male	A	91.4	0.3	G	14.4	0.6	0.49	41038	66.1		
	FCB 17	male	A	96.8	0.3	G	5.0	0.5	0.60	37184	62.5		
	FCB 18	male	A	98.1	1.0	G	5.1	0.7	0.51	22013	66.8		
	FCB 19	female	A	95.9	0.4	G	4.6	1.0	0.53	23356	64.3		
	FCB 20	female	A	97.6	0.8	G	3.7	1.7	0.46	31064	67.9		
	FCB 21	female	A	96.2	2.4	G	3.9	7.9	0.43	22667	68.5		
