# Supplementary material for: Hypermethylation of the non-imprinted maternal MEG3 and paternal MEST alleles is highly variable among normal individuals
Source: PLoS One. 2017 Aug 30;12(8):e0184030. doi: 10.1371/journal.pone.0184030 (PMC5576652; doi:10.1371/journal.pone.0184030)
Supplement: S5 Table — (PDF) [file pone.0184030.s008.pdf]

S5 Table. Epimutation rates measured by DBS.

Gene	Tissue[a]	Sample size (N)	Epimutation rate on the maternal allele (%)[b]				Epimutation rate on the paternal allele (%)[b]			
			Min	Max	Mean	SD	Min	Max	Mean	SD
**Roche GSJunior**										
MEG3 IG DMR	FCB	30	4.6	22.1	13.4	4.3	1.1	12.8	6.6	3.3
	AB	23	10.0	65.5	23.3	11.8	1.9	15.2	5.2	2.8
	VAT	13	7.8	52.1	22.7	11.4	2.8	14.7	6.0	3.5
MEST promoter	FCB	50	0.0	15.4	3.8	4.4	2.1	61.5	18.5	14.3
	AB	36	1.4	9.1	3.3	1.7	2.3	57.6	12.4	10.3
	VAT	24	0.3	3.6	2.1	0.8	1.9	19.8	7.5	4.5
**Illumina MiSeq**										
MEG3 IG DMR	FCB	45	4.9	25.9	13.6	4.4	0.9	11.3	3.1	2.4
MEG3 promoter	FCB	31	0.3	54.5	5.6	10.5	0.1	3.9	1.0	1.0
MEST promoter	FCB	58	0.2	13.6	1.8	2.9	0.7	41.8	18.6	10.9
PEG3 promoter	FCB	21	0.2	6.2	1.5	1.7	0.3	17.3	3.4	5.1

[a] FCB = fetal cord blood, AB = adult blood, VAT = viscertal adipose tissue.
[b] Epimutations are alleles with >50% aberrantly (de)methylated CpG sites.
